# Harnessing synaptic vesicle release and recycling with antibody shuttle for targeted delivery of therapeutics to neurons

**DOI:** 10.1016/j.omtm.2025.101476

**Published:** 2025-04-19

**Authors:** Karen Kar Lye Yee, Junichi Kumamoto, Daijiro Inomata, Naoki Suzuki, Ryuhei Harada, Norihiro Yumoto

**Affiliations:** 1 Jiksak Bioengineering, Inc., Cybernics Medical Innovation Base-A room 322, 3-25-16 Tonomachi, Kawasaki-ku Kawasaki-shi, Kanagawa 210-0821, Japan; 2 Department of Neurology, Tohoku University Graduate School of Medicine, 1-1 Seiryo-machi, Aoba-ku Sendai, Miyagi 980-8574, Japan; 3 Department of Rehabilitation Medicine, Tohoku University Graduate School of Medicine, 1-1 Seiryo-machi, Aoba-ku Sendai, Miyagi 980-8574, Japan

**Keywords:** drug delivery system, synaptic vesicle transmembrane luminal domain, monoclonal antibody shuttle, blood brain barrier, blood spinal cord barrier, intravenous administration, pre-synapse, neuromuscular junction, motor neurons, systemic administration

## Abstract

The effective delivery of therapeutic molecules to neurons are mainly limited by the presence of the blood-brain barrier (BBB) and blood-spinal cord barrier (BSCB), leading to suboptimal therapeutic outcomes in neurodegenerative diseases treatment. This study introduces a neuron-selective drug delivery system that utilizes the synaptic vesicle release and recycling mechanism (SVRM) to overcome these barriers. This delivery system consists of an antibody shuttle that targets SV transmembrane proteins, which enables selective molecule delivery to neurons. We demonstrated that intravenously administered antibodies raised against the luminal domain of synaptotagmin-2 (SYT2) selectively localize to neuromuscular junctions. They were taken up and retrogradely transported to CHAT-positive motor neurons in both the spinal cord and brainstem. Anti-SYT2 antibody delivery of anti-microtubule agent and *MALAT1* gapmer antisense oligonucleotides (ASOs) induces axonal degeneration and *MALAT1* RNA downregulation *in vitro*, respectively. Additionally, intravenous administration of anti-SYT2 conjugated with *MALAT1* gapmer ASOs in mice resulted in the reduction of *Malat1* RNA in targeted cells. This approach circumvents the BSCB, enabling the neuron-selective delivery of therapeutic agents to increase neuronal drug concentrations while minimizing off-target effects in non-targeted cells. Thus, harnessing the SVRM offers a promising strategy to enhance the therapeutic index for neurodegenerative diseases treatment.

## Graphical abstract

**Figure:**
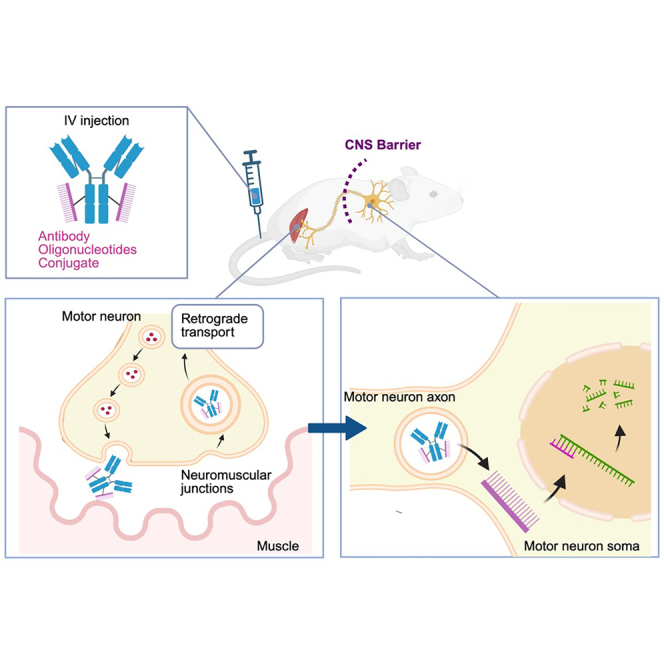

## Teaser

This study presents an antibody-based approach for neuron-selective oligonucleotide delivery. Upon i.v. injection, antibodies targeting SV luminal domains exploit SV recycling to transport oligonucleotides across CNS barriers and into MNs. This strategy enables targeted molecular delivery while minimizing off-target effects, offering a platform for future therapeutic applications.

## Introduction

Developing effective therapies for neurodegenerative diseases remains one of the most challenging and urgent objectives in biomedical research. Key obstacles in this pursuit include the multifactorial pathophysiology nature of these disorders, and the unique barriers at the CNS, namely, the blood-brain barrier (BBB) and the blood-spinal cord barrier (BSCB).^1^^,^^2^ These barriers are composed of specialized endothelial cells, astrocytes, pericytes, efflux transporters, and a basement membrane that form a highly selective interface between the blood and the brain or spinal cord tissue.^3^^,^^4^ While essential for maintaining CNS homeostasis, these barriers present a formidable hurdle in drug development. Estimates suggest that more than 95% of small molecule drugs and nearly all large molecule therapeutics fail to cross the BBB and BSCB effectively.^5^^,^^6^

Hence, overcoming these barriers is crucial for advancing new therapeutics for neurodegenerative diseases. Various mechanisms utilizing receptor-mediated transcytosis, adeno-associated virus, and cell-penetrating peptides have been explored to circumvent the BBB and BSCB for therapeutic molecule delivery to cerebrospinal tissues.^7^^,^^8^^,^^9^^,^^10^ Among these, receptor-mediated transcytosis with engineered antibodies targeting the transferrin receptor has shown some promise enabling therapeutic molecules transcytosis across the BBB.^11^^,^^12^^,^^13^ However, it lacks cell specificity within CNS tissues and the mechanism to transverse neuronal cell membrane after BBB entry.

The therapeutic efficacy of neurological treatments hinges not only on successful drug delivery to CNS but also on precise drug targeting to specific cell types within the CNS. The absence of cell-specific targeting may result in suboptimal drug concentrations in targeted cells or off-target effects on non-targeted cells.^14^ Drug efficacy can also be constrained by insufficient or the absence of efficient intracellular delivery systems to deliver drugs into specific cellular compartments of the targeted cell.^15^ This is particularly relevant for neurodegenerative conditions such as amyotrophic lateral sclerosis, Alzheimer’s disease, and Parkinson’s disease, where specific neuronal populations are affected. To address these challenges, we focused on harnessing the synaptic vesicle release and recycling mechanism (SVRM) by utilizing an engineered antibody shuttle against the SV transmembrane proteins of neurons for therapeutic molecule delivery. This approach provides a means to transport molecules into cells without inducing non-physiological activities, such as receptor-mediated endocytosis observed in typical antibody drug conjugate in oncology, and it ensures neuron selectivity post-BBB or -BSCB transversal since SVRM is a unique feature for neurons.^16^

In this study, synaptotagmin-2 (SYT2) was selected to illustrate monoclonal antibodies raised against luminal domains of SV transmembrane proteins are capable of targeted molecule delivery to neurons through SVRM. Our immunohistological analyses demonstrate that the intravenously administered monoclonal SYT2 antibody shuttle (mAb-SYT2) was selectively and efficiently taken up by motor neurons (MNs) at the neuromuscular junctions (NMJs) and retrogradely transported to the soma in the spinal cord and brainstem. Furthermore, the *in vitro* and *in vivo* data show that *MALAT1* gapmer antisense oligonucleotides (ASOs) conjugated with mAb-SYT2 reduces *MALAT1* RNA expression in targeted cells. This indicates that payloads delivered by mAb-SYT2 were successfully released into MNs cytoplasm.

Here we propose that antibodies shuttle targeting luminal domain of SV transmembrane proteins can utilize SVRM as an alternative route to deliver therapeutic molecules to intended neuronal populations.

## Results

### LRRTM2-coated microbeads induces SVRM in human iPSC-derived MNs

The conventional method of delivering therapeutic molecules to neurons is through intrathecal injection, which causes adverse effects in patients.^17^^,^^18^

Here we propose a different route to deliver molecules by utilizing SVRM occurring at the pre-synapses. To validate this concept, as well as to acquire high-affinity antibodies that can target the luminal domains of SV transmembrane proteins and be efficiently internalized, an *in vitro* induced pre-synapse model was developed. The *in vitro* induced pre-synapse model uses microbeads coated with leucine-rich repeat transmembrane protein 2 (LRRTM2), a purified postsynaptic membrane protein, to initiate pre-synapse differentiation in human induced pluripotent stem cell (iPSC)-derived MNs. As previous studies have shown that overexpression of synaptogenic transmembrane proteins in fibroblasts or coating microbeads with fabricated clusters of synaptogenic extracellular domains (ECDs) proteins can induce pre-synaptic differentiation in cultured neurons.^19^ We postulate that coating microbeads with LRRTM2 synaptogenic ECD will also induce human iPSC MN for pre-synaptic differentiation in the *in vitro* induced pre-synapse model. LRRTM2 is one of the well-characterized postsynaptic proteins known to induce pre-synaptic differentiation in hippocampal and cortical neurons in mice.^20^^,^^21^^,^^22^^,^^23^^,^^24^

**Figure 1:**
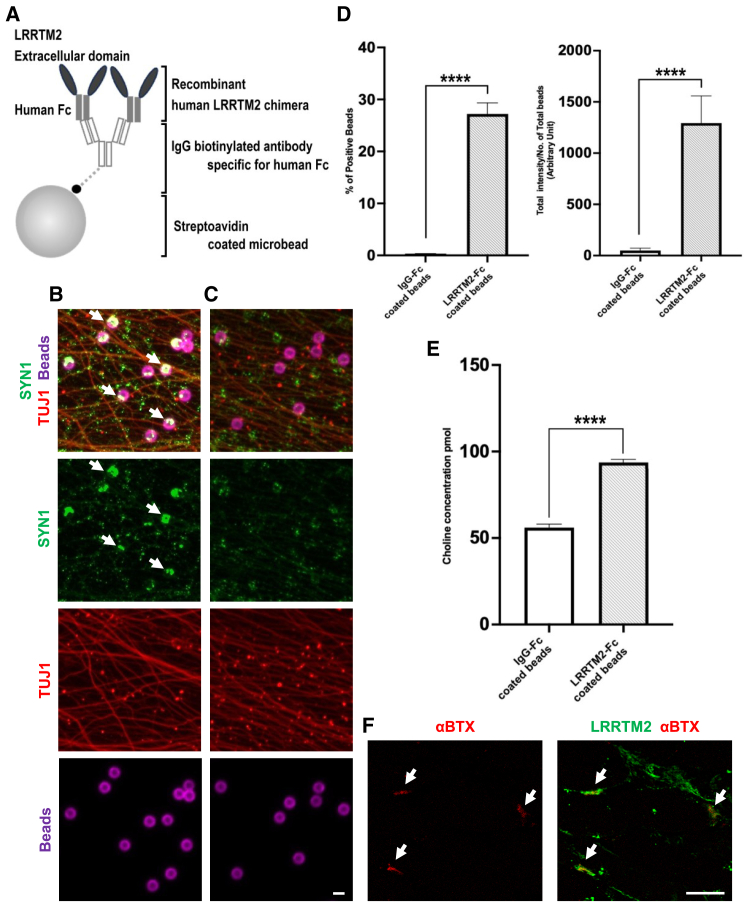
Establishment of an *in vitro* synaptogenesis model using iPS-derived MNs (A) Diagrammatic representation of pre-synapse induction microbeads coated with human LRRTM2 protein. (B) The addition of LRRTM2-coated microbeads induces pre-synapse differentiation (yellow, white arrows) with overlapping expression of SYN1 pre-synapse marker (green, white arrows), TUJ1 axon marker (red), and the presence of LRRTM2-coated microbeads (magenta). (C) Minimal pre-synapse differentiation with control IgG-coated microbeads. (D) Quantification of pre-synapsed formed (left) and density of pre-synapse per bead (right), *n* = 5. (E) Acetylcholine was converted to choline for total choline amount detection with the assay kit. Medium from the axon well of nerve organoid cultured in B-type microchip were stimulated with pre-synapse-induced microbeads and SV exocytosis stimulation with 4AP had functional increased in acetylcholine secretion compared with stimulation with control IgG-Fc microbeads and 4AP (*n* = 4). (F) Localization of LRRTM2 at the NMJ in human skeletal muscle, α-bungarotoxin (top) and co-localization of LRRTM2 (labeled in green) with α-bungarotoxin (labeled in red), arrow indicates NMJ (bottom). Scale bars, (C) 10 μm and (F) 50 μm. All data expressed as mean ± SEM and t test analysis; ∗∗∗∗*p* < 0.0001.

Our findings demonstrated that microbeads coated with LRRTM2 ECDs fused to human immunoglobulin G (IgG) Fc domain were able to induce human iPSC-derived MNs to form synapsin-1 aggregates at microbeads contact sites, indicating pre-synaptic differentiation of cultured neurons. Control human IgG-Fc-protein-coated microbeads had no synapsin-1 aggregation effect at microbeads (Figures 1A–1D). The differentiated pre-synapse, upon stimulation with 4-aminopyridine (4AP) had increased acetylcholine secretion detected in the culture medium but not with neurons treated with control IgG microbeads (Figure 1E). These data indicate that LRRTM2 induces pre-synaptic differentiation in human MNs. Furthermore, our immunostaining experiments on human muscle specimens revealed an accumulation of LRRTM2 proteins at NMJs, suggesting a functional role for LRRTM2 in formation and/or maintenance of human NMJs (Figure 1F).

**Figure 2:**
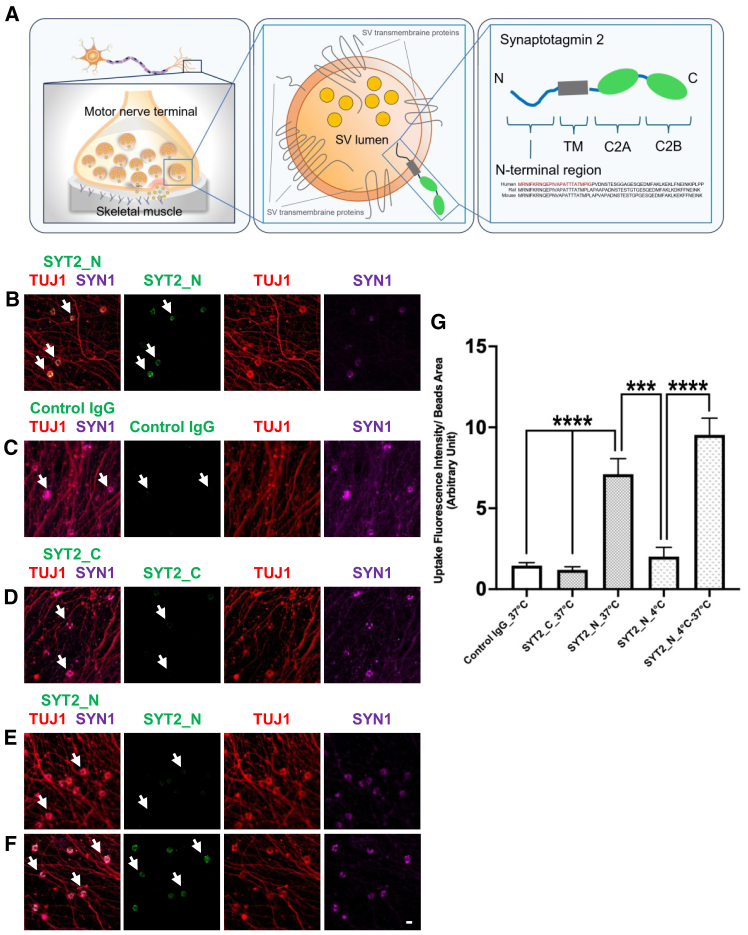
Antibody shuttle utilizes SVRM in human iPSC-derived MN for internalization (A) SYT2 luminal domain targeted by antibodies when it is exposed to the extracellular space during SV exocytosis. TM, transmembrane domain. C2A and C2B are calcium sensor domains located in the cytoplasm. (B) Antibody shuttling through SVRM requires antibody specificity to the luminal domain of SV transmembrane protein. Luminal domain targeting antibody, SYT2 N-terminal, localizes to pre-synapse on LRRTM2-coated microbeads (yellow, white arrows). (C and D) Absence of pre-synapse localization with C-terminal antibodies targeting the cytoplasmic domain of SYT2 and control IgG (arrows). (E) Exo-endocytosis is required for antibody shuttling into SVRM. At 4°C, there is a decrease in pre-synapse exocytosis activity with no localization of SYT2 N-terminal antibodies to the pre-synapse (arrows). (F) Recovery of exocytosis when reverted to 37°C. Localization of SYT2 N-terminal antibodies to the pre-synapse (green, white arrows) and merged images (yellow, white arrows). (G) Graphical representation of antibodies uptake at pre-synapse (D-H), *n* = 4. Scale bar, 10 μm. All data are expressed as mean ± SEM and one-way ANOVA analysis; ∗∗∗∗*p* < 0.0001, ∗∗∗*p* < 0.001.

The suitability of antibody shuttles for SVRM needs to be characterized with a high selectivity and affinity toward the luminal domain of SV transmembrane proteins, which are transiently exposed during SVRM cycles. In order to identify that antibody binding to the luminal domain during SVRM is crucial for this delivery system, we used the *in vitro* induced pre-synapse model and commercially available anti-SYT2 N-terminal (luminal domain) rabbit polyclonal antibody for antibody uptake study, illustrated in Figure 2A. Neuronal stimulation with 4AP at 37°C showed SYT2 antibody internalization (Figure 2B). In contrast, control normal rabbit IgG and anti-SYT2 C-terminal (cytoplasmic domain) rabbit polyclonal antibodies were not internalized when stimulated with 4AP at 37°C (Figures 2C and 2D). To demonstrate that the antibody uptake depends on SVRM and not the presence of 4AP, we investigated the temperature dependency required for antibody internalization. At 4°C, the reduction of exo-endocytotic activities inhibits SYT2 N-terminal antibody uptake (Figure 2E). When the iPSC-derived MNs were reverted to 37°C, anti-SYT2 N-terminal antibody localization to the SV was again observed (Figure 2F). This suggests that SVRM antibody shuttle ability to deliver therapeutic molecules to neurons, which is dependent on the neuronal exo-endocytosis process and a specificity for SV luminal domain, is feasible.

### Generation and characteristics of monoclonal antibodies targeting luminal domains of SV transmembrane protein

After conceptualizing SVRM as a potential route for antibody shuttle uptake, monoclonal antibodies that had high specificity for luminal domain of the SV transmembrane protein were required. Several transmembrane proteins localized in the SV are involved in neurotransmission. Through published literature and public domain databases, 16 such potential candidates were identified; we selected SYT2 to exemplify our concept further. SYT2 being a single-pass membrane protein allows the ease of monoclonal antibody generation.^25^ Furthermore, its main physiological function in calcium sensing and the SV recruitment mechanism to the active zone for neurotransmitter release is located at the cytoplasmic C terminal region consisting of 336 amino acid (aa) residues. We postulate that generating antibodies against the luminal domain, full 62 and partial 25 aa residues, will have the minimum physiological hindrance. In addition, SYT2 accumulates at all NMJs whereas SYT1 its closest homology among 17 synaptotagmin family members accumulates at less than 50% of NMJs in mice and rats.^26^^,^^27^ Thus, SYT2 monoclonal antibodies are described here, while the rest of the generated antibodies against other SV proteins antigens are not shown (Table S1; Figure S1).

Based on fluorescence-activated cell sorting (FACS) and ELISA screening, primary and secondary candidates were identified from 37 scFv clones against human SYT2 full N-terminal domain (h2FL,1–62 aa) and 93 clones against the partial N-terminal domain (h2PL,1–25 aa) were allocated into 7 clusters each. These scFvs are reactive to both human SYT2 and mouse SYT2, but not to human SYT1 (data not shown). For final selection, we examined the binding and internalizing capabilities of these scFv clusters with the *in vitro* induced pre-synapse model. Two h2FL and four h2PL were identified to efficiently internalize into pre-synaptic specializations induced by synaptogenic microbeads on human iPS-derived MNs in the presence of 4AP (Figures S1B and S1C). The characteristics of these six monoclonal SYT2 scFvs are summarized in Table S1. Selected anti-SYT2 scFvs were converted to chimeric full-length IgGs with human Fc regions (mAb-SYT2). Hereafter, all data shown in this report were collected by using chimeric mAb-SYT2 clone against full luminal domain and partial luminal domain, unless otherwise described.

Dissociation constant (K_D_) for these six mAb-SYT2 was determined. Since there was no commercially available monoclonal antibody that is reactive to human SYT2, we used the K_D_ of FL01 converted to chimeric full-length IgG to benchmarked the six antibodies K_D_. Our data show that FL01 falls within the median cutoff range in the candidate selection uptake study (Figure S1B). A ratio higher than FL01 indicates lesser SYT2 antigen affinity and a lower ratio indicates greater SYT2 antigen affinity (Table S2). PL13 showed a median ratio value of 1.7 and was selected for subsequent studies.

### *In vivo* uptake and distribution of SYT2 antibody shuttle

**Figure 3:**
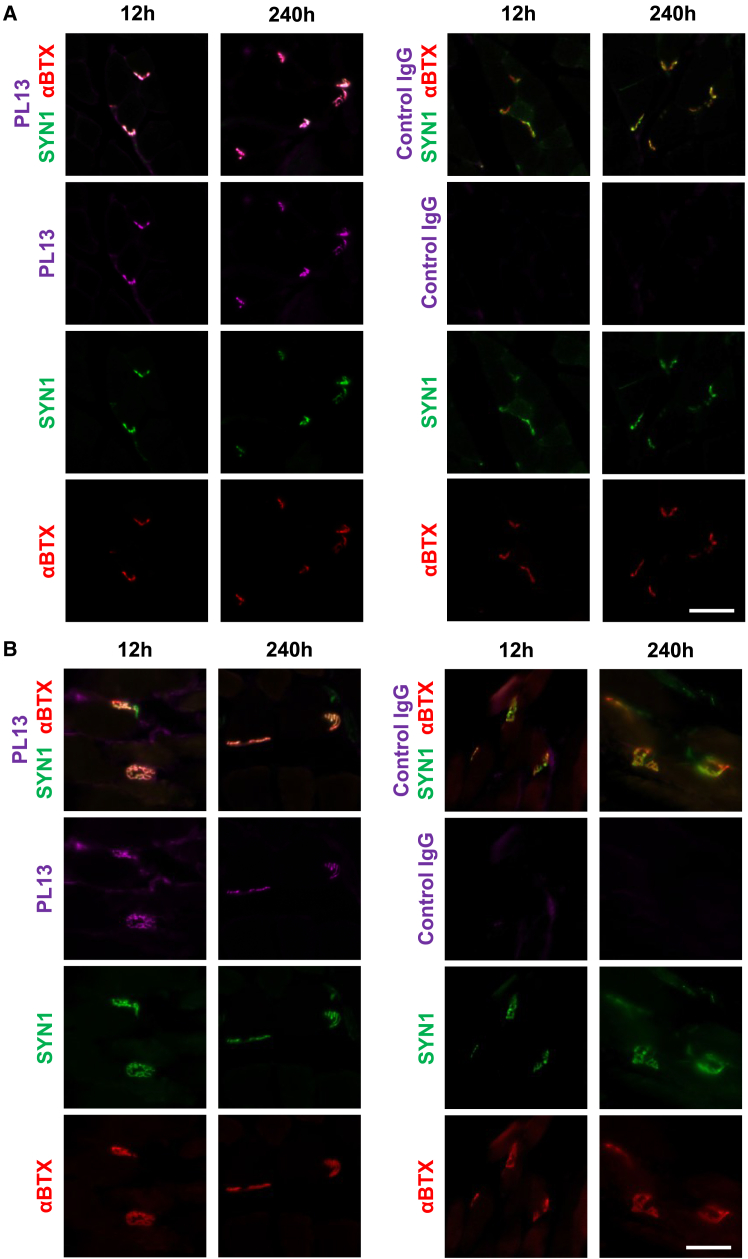
NMJ uptake of SVRM antibody shuttle after i.v. administration (A) PL13 uptake at NMJ of gastrocnemius (GAS) tissues were observed at 12–240 h (magenta, left) but not with control IgG at 12–240 h (right). Scale bar, 50 μm. (B) PL13 uptake at NMJ of tibialis anterior (TA) tissues were also observed (magenta, left) from 12 to 240 h but not with control IgG antibodies (right). Scale bar, 50 μm. The 24-h and 72-h images are available in Figure S2 showing the same trend. See also Figures S6 and S7.

Neuronal uptake of PL13 upon intravenous (i.v.) injection at various time points in mice was investigated by immunostaining with fluorophore-conjugated anti-human IgG Fc domain antibodies. The PL13 signals were localized at NMJs, which were co-visualized with synapsin-1 antibody and α-bungarotoxin, from 12 h to 240 h after i.v. injection in gastrocnemius and tibialis anterior muscle tissue (Figures 3A, 3B, and S2, left). As PL13 is taken up at the NMJ, it is also retrogradedly transported into the axon (Figures S6 and S7). Random localization of control IgG antibodies at NMJs was of undetected amount at all time points and no retrograde transport into axon was observed (Figures 3A, 3B, S2 right, S6, and S7).

**Figure 4:**
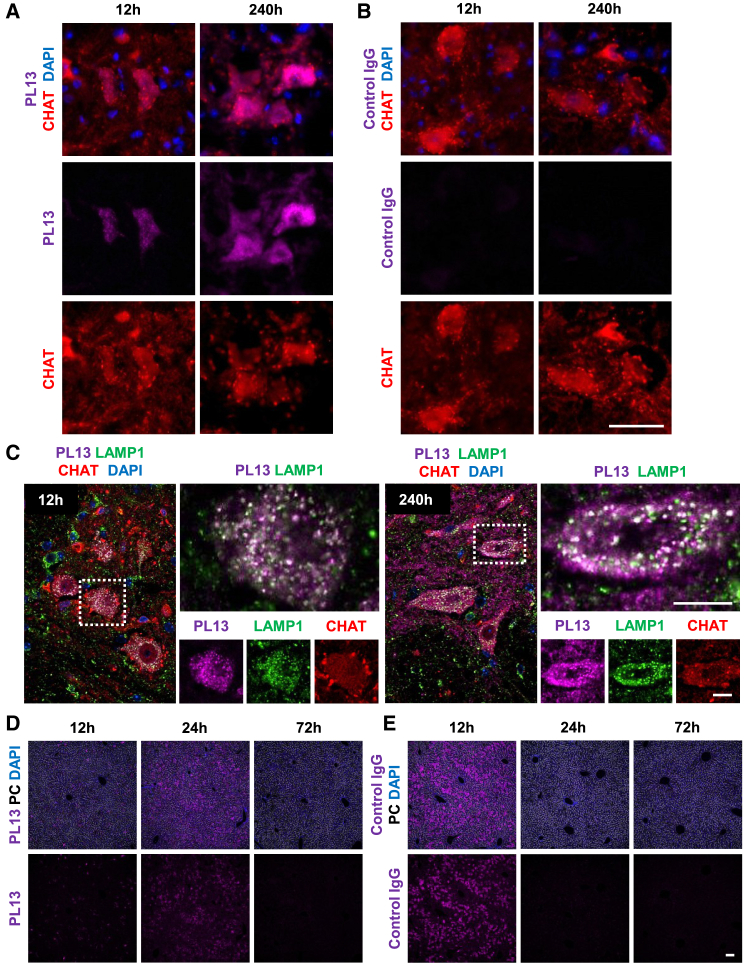
*In vivo* distribution of SVRM antibody shuttle after i.v. administration (A) At 12 and 240 h after NMJ uptake of PL13 (magenta), antibodies retrograde to MN soma are located in the ventral horn of the spinal cord. At 240 h, PL13 was intensely localized to CHAT-stained MN (red). (B) Absence of control IgG at MN soma at 12 and 240 h. Scale bar, 50 μm. See also Figure S3A. (C) Detection of PL13 (magenta) in lysosomes (Lamp1, green) of MN soma from 12 to 240 h. Indicates the shuttle is retrograded to the spinal cord through the endo-lysosome system after SVRM. Scale bar, 10 μm. (D and E) Uptake and clearance of PL13 in liver. (E) Partial luminal domain (PL) 13 uptake peaks at 24 h and cleared by 72 h. (E) Control IgG uptake peaks at 12 h and cleared by 72 h. Scale bar, 100 μm. (See also Figure S3).

To maintain neuronal homeostasis, it is essential for damaged, aging proteins and organelles, including SVs, to be removed from the axon terminals and distal axon by retrograde transport to the soma for degradation and recycling of components. Hence, we investigated the distribution of PL13 antibodies to the soma. Frozen sections of the spinal cords at the lumbar region from the same animals used in NMJ localization experiments were co-immunostained with CHAT antibody and anti-human IgG Fc antibody in the soma. Weak signals of PL13 were observed at 12–24 h and accumulated intensely at 72–240 h in CHAT-positive MNs located at the lumbar region of lateral ventral horn of the spinal cord (Figures 4A and S3, left). Control IgG antibodies in CHAT-positive MNs were undetected at all time points (Figures 4B and S3, right). Interestingly, the subcellular localization of PL13 signals in the soma were co-stained in lysosome with LAMP1 and some signals outside of the lysosome (Figure 4C). This suggests that endosomes and/or autophagosomes generated from the post-SVRM uptake of PL13 underwent cargo sorting and were transported to the soma for protein degradation or recycling.

In the liver, control IgG and PL13 peak detection is at 12 and 24 h, respectively; by 72 h, both antibodies were not detectable in the liver (Figures 4D and 4E). This indicates that there is no difference in the antibody clearance rate between mAb-SYT2 and control IgG in the liver.

**Figure 5:**
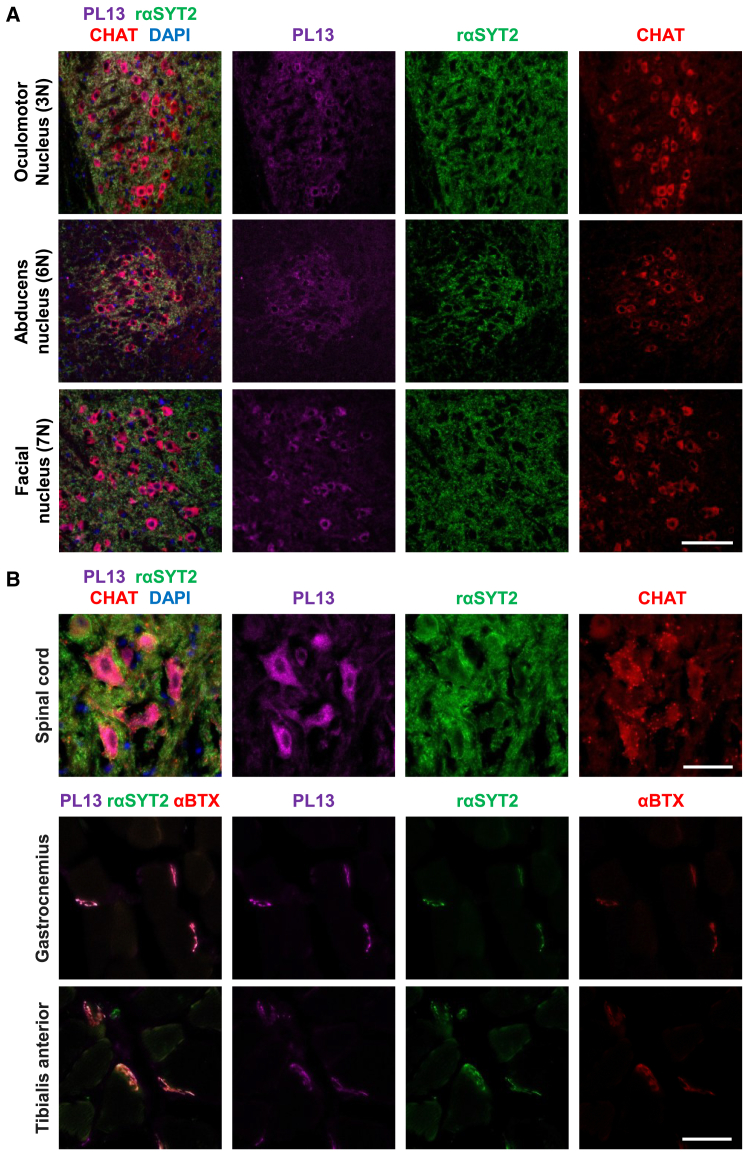
SVRM antibody shuttles distribution in brainstem, spinal cord and NMJ after i.v. administration with respect to intrinsic SYT2 expression Uptake of (A) localization of PL13 (magenta) to brainstem MNs at 3N, 6N, and 7N, (B) spinal cord and NMJ at 240 h after i.v. injection had co-localization with CHAT-stained (red) and intrinsic expressing SYT2 (r*α*SYT2, green). Scale bar, (A) 100 μm and (B) 50 μm. (See also Figures S4 and S5).

Most cranial MNs originate from the brainstem and innervate peripheral muscles in the head and neck region, controlling functions such as eye movement, facial expression, mastication, swallowing, and speech. Subsequently, PL13 uptake through SVRM and retrograde transport to the soma in the cranial MNs was examined. At day 10 after i.v. administration, brainstem frozen sections were analyzed with immunostaining. Visualization of PL13 revealed its localization in CHAT-positive somas of the oculomotor nucleus (3N), abducens nucleus (6N), and facial nucleus (7N) (Figure 5A). Intriguingly, the staining of intrinsically expressing SYT2 with rabbit polyclonal anti-SYT2 in the brainstem did not overlap with the PL13 signals. This indicates that PL13 after uptake localizes to regions different from intrinsic expressing SYT2 regions or that there could be SYT2 competitive binding. In the spinal cord, there is some overlap of PL13 with intrinsic expressing SYT2. At the NMJ, PL13 converged with intrinsic expressing SYT2 (Figure 5B). Indicating that, after PL13 uptake at the NMJ, there is a decoupling of PL13 from SYT2 antigen as it gets transport away from the NMJ. Control IgG antibodies were not detected in the brainstem at all time points (data not shown).

**Figure 6:**
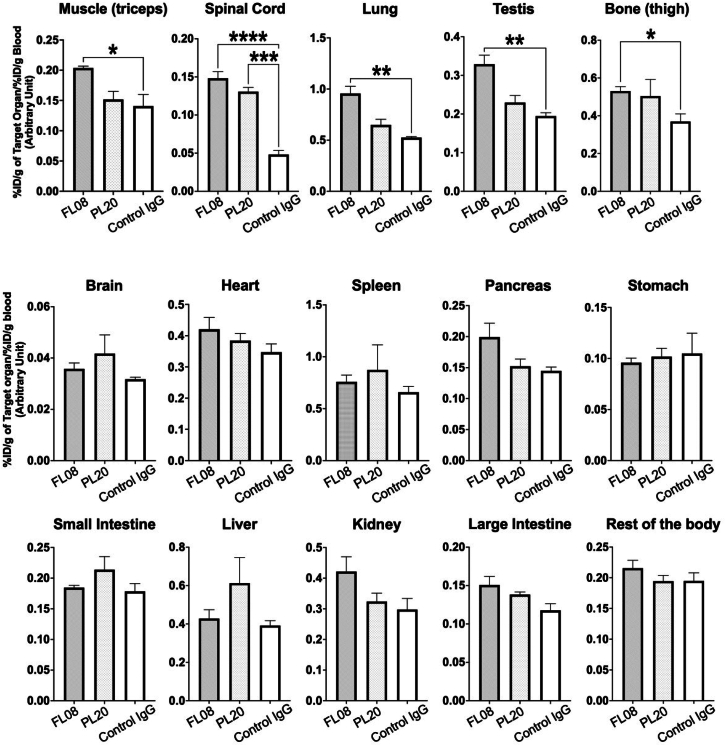
*In vivo* distribution of FL08 and PL20 were conjugated with RI Zr^89^ FL08-Zr^89^ significantly localizes to muscle, lung, bone, and testis compared with control IgG-Zr^89^. Both FL08 and PL20 substantially retrogrades more Zr^89^ to the spinal cord compared with control IgG-Zr^89^. Data expressed as mean ± SEM, *n* = 3 and one-way ANOVA analysis; ∗∗∗∗*p* < 0.0001, ∗∗∗*p* < 0.001, ∗∗*p* < 0.01, ∗*p* < 0.05. (See also Table S3).

mAb-SYT2 shuttle distribution and uptake were further investigated with FL08 and PL20 conjugated with zirconium radioisotope (RI). At 72 h, the uptake of mAb-SYT2 was quantified with a localization ratio that factors in blood clearance of the antibodies by determining the ratio of percentage of injected dose per gram (%ID/g) in organ and blood. The localization ratio showed FL08 was significantly detected in the lung, testis, muscle (triceps), and bone (thigh) compared with control IgG (Figure 6).^28^

Both FL08 and PL20 exhibited significantly higher spinal cord localization ratios (Figure 6). To grasp the selectivity and efficacy of mAb-SYT2 tendency to retrograde to the spinal cord, we evaluated the immunospecificity index of a specific organ by dividing localization ratio of mAb-SYT2 against the control. Both FL08 and PL20 had a 2.5- to 3-fold immunospecificity index in the spinal cord after i.v. injection (Table S3). This is noteworthy given that whole IgG antibodies have difficulty crossing the BSCB. The observed spinal cord penetration suggests antibodies shuttle utilizing SVRM properties at spinal MNs were able to achieve CNS entry.

### *In vitro* and *in vivo* delivery of an active payload by mAb-SYT2

**Figure 7:**
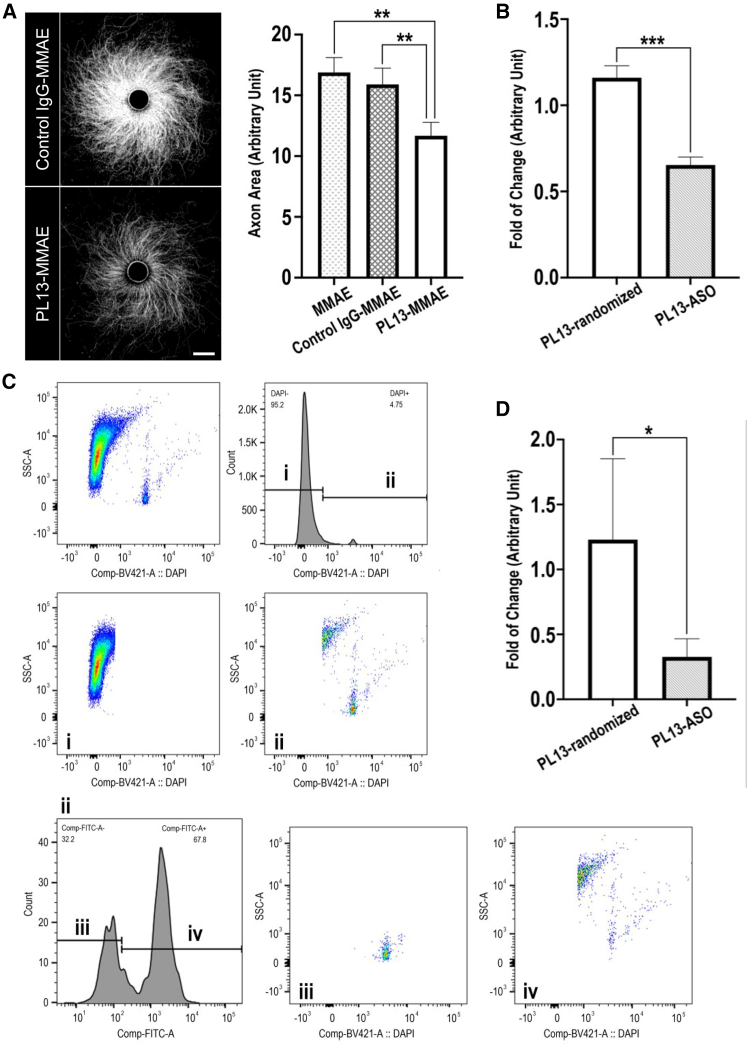
Delivery of active payloads to MN with SVRM antibody shuttle (A) (Left) Representation of PL13-MMAE delivery resulted in axon degeneration observed in the *in vitro* model. Scale bar, 500 μm. (Right) Graphical representation of PL13-MMAE delivery in the *in vitro* model showed 26.6% decrease in axon area compared with control chimera IgG-MMAE. Both MMAE alone and IgG-MMAE have similar axon areas, indicating active delivery of MMAE into MNs by PL13 resulting in axon degeneration. (B) *In vitro* delivery of PL13-*MALAT1*-ASO resulted in 34% knockdown of *MALAT1* RNA compared with delivery of PL13-randomized-ASO. (C) Mice were intravenously injected with PL13 conjugated with *MALAT1*-ASO or randomized-ASO. Cells were gated for weak to strong DAPI staining, (i) DAPI-negative cells (ii). DAPI-positive cells, (ii) were further sorted for PL13-positive (iv) or negative (iii) populations. (D) PL13-positive cells from PL13-*MALAT1*-ASO injected mice had 73.4% RNA knockdown compared with PL13-positive cells from PL13-randomized-ASO injected mice. Data are expressed mean ± SEM (*n* = 3) and (A) analyzed with one-way ANOVA, (B and D) analyzed with t tests. ∗∗∗*p* < 0.01, ∗∗*p* < 0.05, ∗*p* ≤ 0.1.

We next investigated mAb-SYT2 capability to deliver functional payloads into MNs in the *in vitro* induced pre-synapse model. PL13 was conjugated with an anti-mitotic agent, monomethyl auristatin E (MMAE) and added to cultured MNs. Specific targeting by PL13 and 4AP stimulation, functional MMAE was transported into MNs resulting in axon degeneration (Figure 7A, left). A decrease of 26.6% in axon area (11.67 ± 1.096 SEM) was observed in PL13-MMAE compared with random uptake of control chimera IgG-MMAE (15.9 ± 1.332 SEM) and neurons treated with MMAE only (16.88 ± 1.096 SEM) (Figure 7A, right). The data showed mAb-SYT2 as a potential SVRM shuttle that can transport functional molecules into neurons.

In addition to MMAE, the delivery of ASO to neurons was examined. We constructed *MALAT1* gapmer ASO (*MALAT1*-ASO) with DBCO-PEG4-Val-Cit-PAB-PNP linker. This linker construction with *MALAT1*-ASO neither inhibited ASO activity (Figure S8A) nor did conjugation of PL13 with linker-ASO obstruct mAb-SYT2 binding efficiency to SYT2 antigen (Figure S8B). The conjugation rate of PL13 with linker-ASO was approximately 80% based on band intensity analysis with ImageJ2 software, version 2.14.0 (Figure S8C).

Utilizing the same *in vitro* induced-pre-synapse model, PL13 conjugated with 0.6 μM *MALAT1*-ASO had a 34% decrease (0.6533 ± 0.0464 SEM) in *MALAT1* RNA expression compared with control PL13-randomized-ASO (1.160 ± 0.0697 SEM) (Figure 7B). These results showed that mAb-SYT2 are capable of transporting functional small molecules and oligonucleotides into neurons.

Furthermore, we sought to determine whether mAb-SYT2 was able to deliver functional ASO *in vivo*. Mice were intravenously injected with PL13-*MALAT1*-ASOs or PL13-randomized-ASOs had the spinal cords collected on day 10 after i.v. injection for *Malat1* RNA quantification. Because MNs are such a small population in the spinal cord, we isolated PL13-labeled cells from the spinal cord through cell sorting, which was achieved by additional PL13 i.v. administration at day 7 before sample collection at day 10. Spinal cord cells stained for DAPI were gated with FACS. PL13-positive cells were sorted by fluorescence staining for human Fc region of PL13 (Figure 7C). PL13-positive cells isolated from mice injected with PL13-*MALAT1*-ASO had a notable reduction in *Malat1* RNA expression (0.327 ± 0.1398 SEM) compared with PL13-positive cells of mice injected with PL13-randomized ASOs (1.23 ± 0.6228 SEM) (Figure 7D). Similarly, PL13-positive cells showed a decrease in *Malat1* RNA expression compared with PL13-negative cells isolated from the spinal cord of mice injected with PL13-*MALAT1*-ASOs (Figure S8D). These results indicate that PL13 delivery of *MALAT1*-ASOs was able to escape from the endo-lysosomal system into the cytoplasm and enter the nucleus for successful knockdown. As a valine-citrulline linker, that is enzymatic cleavable, was used to conjugate PL13 and *MALAT1*-ASOs. After cleavage, *MALAT1* ASO is likely to escape from the endo-lysosomal system during membrane fusion processes; however, the exact mechanisms underlying these processes remain unclear.^29^ Together, the *in vitro* and *in vivo* data signify that the SVRM shuttle is capable of delivering therapeutic molecules to neurons. Specifically, when SYT2 is used as an antigen and administered intravenously, mAb-SYT2 can deliver therapeutic molecules through the MNs to the spinal cord.

## Discussion

This study demonstrated the capacity of antibodies as SVRM molecular shuttles that target the luminal domain of SV transmembrane protein are capable of delivering small molecules and oligonucleotides to neurons. We used spinal MNs as a model system, capitalizing on their axon terminals are exempted from the BBB and BSCB regulation. This model has *in vivo* spatial separation of MNs somata located inside of these barriers and the synaptic terminals on the outside of these barriers. Our focus centered on SYT2, a protein abundantly expressed in skeletal muscle-projecting neurons, whose single-pass membrane structure renders it ideal for antibody generation.^30^^,^^31^

Our findings reveal that the systemic administration of mAb-SYT2 results in SVRM-mediated uptake at the axon terminal, analogous with botulinum neurotoxin (BoNT) transport mechanisms.^32^ In the spinal cord, mAb-SYT2 signals accumulate in CHAT-positive MNs, suggesting retrograde transport of antibody-containing SVs via endosomal and/or autophagosomal pathways, as evidenced by the co-localization of the lysosomal marker LAMP1 in the soma.

Extending our observations to cranial MNs, we detected mAb-SYT2 signals in CHAT-positive brainstem nuclei, including the oculomotor, abducens, and facial nuclei. Interestingly, the majority of intrinsically expressing SYT2, visualized with a polyclonal antibody, did not colocalize with mAb-SYT2 signals, suggesting possible SYT2 competitive binding. However, the data more likely indicate that post-uptake mAb-SYT2 localizes differently compared with intrinsic axonal SYT2 localization.

Our data also showed that mAb-SYT2 utilizing SVRM was capable of delivering functional molecules into MNs and retrograde to the spinal cord. Miyashita et al.^33^ reported the usage of deactivated BoNT for peptide delivery into MNs, but there remain risks of residual toxicity and it may induce the production of neutralizing antibodies due to its immunogenicity.^33^^,^^34^ Instead, the use of antibodies for therapeutic molecule delivery can circumvent these issues as antibodies are well-characterized molecules, host compatible, and their clinical safety profiles are well established in various conditions such as long-term multiple administrations.^35^^,^^36^^,^^37^

A critical challenge in neurodegenerative disease treatment and its development is to efficiently transport therapeutic molecules into the cerebrospinal tissue, where molecular permeability is tightly regulated by the BBB and BSCB. In recent years, transferrin receptor-mediated transcytosis and intrathecal injection have attracted attention to address these challenges. However, both approaches lack cell specificity once inside the BBB and BSCB.^38^^,^^39^^,^^40^ This potentially results in suboptimal therapeutic concentrations in target cells and raises the risk of adverse reactions in non-targeted cells.^41^

Additionally, intrathecal injection is invasive and burdensome for patients.^42^ In contrast, systemic administration of SV lumen-selective antibodies, for example, mAb-SYT2, could overcome these limitations to target MNs in the spinal cord and brainstem. Thus, i.v. injection of mAb-SYT2 could serve as a molecular shuttle to spinal and brainstem MNs.

The principle underlying our approach extends to other SV transmembrane proteins. For example, synaptophysin, the most abundant SV protein, presents an opportunity for potentially enhanced molecule targeting and delivery to spinal MNs. Furthermore, the administration of SV lumen-selective antibodies by intrathecal injections or engineering bispecific antibodies targeting transferrin receptors might allow specific targeting to distinct neurons based on SV proteins expression profile in targeted neurons after BBB entry.

In conclusion, antibody shuttle targeting SYT2 luminal domain demonstrates the feasibility of employing SV lumen-selective antibodies as neuron molecular shuttles via SVRMs. Various neuronal subtypes express different SV transmembrane proteins by engineering antibodies with diverse features, consisting of affinity to a defined luminal domains of SV transmembrane proteins; thus, we can achieve precise targeting of neurons while minimizing off-target effects. This can pave the way for new possibilities of developing tailored therapeutic molecule delivery systems, as well as targeted interventions to specific neurons. This will allow more effective and personalized treatments for neurological disorders. Future research in this direction could lead to a new generation of neuron-specific drug delivery platforms, potentially revolutionizing the field of neuropharmacology and improving patient outcomes.

### Limitations of the study

SYT2 expression is not confined to spinal MNs; it is also prominent in the brainstem, as corroborated by our immunostaining data and literature reports.^43^ Antibodies can migrate into cerebrospinal tissues after i.v. administration, indicating that both the target antigen and the administration method significantly influence cell selectivity.^44^ The mechanism of antibody transcytosis into CNS cells post-NMJ retrogradation requires further investigation to elucidate therapeutic molecule distribution when using mAb-SYT2 as a shuttle. Our data confirm mAb-SYT2 presence in endo-lysosomes, which might imply cellular stress or physiological inhibition risks, although no immediate adverse effects were observed in *in vivo* studies. Comprehensive risk management remains crucial during drug development, necessitating detailed studies to ensure safety and efficacy.

## Materials and methods

### Animals

For antibody distribution and ASO studies, we used -5 to 8-week-old female C57Bl/6 mice, and for RI studies, we used 5- to 7-week-old male ICR mice. All mice were purchased from Jackson Laboratory Japan Co. Mice were housed in a temperature- (22°C–24°C) and humidity- (40–51%) controlled room. Food and water were provided *ad libitum*, and the mice were housed under a 12-h light/dark cycle. All behavioral manipulations were performed during the light phase. We complied with local and national ethical and legal regulations regarding the use of mice in research. All experimental protocols were approved by the animal testing regulations of KAC Co, Nihon Medi-Physics Co, the animal experiment committee regulations, and the animal experiment approval regulations.

### Human samples

The human study protocol was approved by the Tohoku University Hospital’s Institutional Review Board (Approval nos. 2022-1-144, 2022-1-848).

### Cells lines

HEK293T cells (CRL-3216, Lot:70041165) were purchased from ATCC. iPSC-derived normal human MNs (Lots: 200238, 400329, and 400445) were purchased from iXcells. Cultures were performed as described in method details.

### Method details

#### Preparation of synaptic microbeads

The preparation of synaptic microbeads was based on Yumoto et al.^19^ Briefly, SuperAvidin Coated Microspheres (Beads, Bangs Laboratories) were aliquoted at a concentration of 200 μg/mL and then washed with wash buffer (0.02% albumin, BSA [NACALAI TESQUE] in PBS [WAKO]). Anti-human IgG (Fc-specific)-biotin antibody, mouse monoclonal (Sigma Aldrich Technologies) at a concentration of 111 μg/mL was added, and then the microbeads were inverted and mixed at 4°C for 3 h and then washed with wash buffer. Synaptic microbeads were added with Recombinant Human LRRTM2 Fc chimera protein, CF (R&D Systems) at a concentration of 66.6 μg/mL. Control microbeads were added with native human IgG FC fragment protein (Abcam) at a concentration of 66.6 μg/mL. Microbeads were mixed and inverted at 4°C for 3 h and then washed with wash buffer. The final concentration of beads was 200 μg/mL.

#### Cell culture (MNs)

Human MNs (iXCells) were thawed and processed with Dead Cell Removal Kit (STEMCELL Technologies) according to manufacturer’s protocol. Cells were seeded onto a PrimeSurface Plate96V (Sumitomo Bakelite) at 2 × 10^4^ cells/well. Then, the cells were incubated at 37°C and 5% CO_2_ for 1 week to produce spheroids. During the cell culture, the medium was replaced twice using the MN Culture Medium (iXCells). For 96-well plates, spheres were transferred from PrimeSurface Plate 96V to EZVIEW Glass Bottom Culture Plates LB (IWAKI) that were coated with 10 μg/mL poly-D-lysine hydrobromide (Sigma-Aldrich) for 1 day at 4°C and then coated with 175 μg/mL Geltrex LDEV-Free, hESC-Qualified, and Reduced Growth Factor Basement Membrane Matrix (Thermo Fisher Scientific) for 3 h at 37°C with 5% CO_2_. For B-type microchip (Jiksak), spheres were transferred from a PrimeSurface Plate 96V to a B-type microchip that was coated with a mixture containing 12.5 μg/mL laminin (R&D Systems, Cat#3400-010-02) and 180 μg/mL Geltrex LDEV-Free, hESC-Qualified, Reduced Growth Factor Basement Membrane Matrix in DMEM/F12 medium (Thermo Fisher Scientific) for 1 h at 37°C with 5% CO_2_. The cells were cultured for 1 month, changing the medium three times a week. Cells were cultured in Neurobasal Plus Medium (Thermo Fisher Scientific), 2% B-27 Plus Supplement (50×) (Thermo Fisher Scientific), 1% penicillin-streptomycin (Thermo Fisher Scientific), 20 ng/mL brain-derived neurotrophic factor (BDNF, GenScript), 20 ng/mL glial cell line-derived neurotrophic factor (GDNF, GenScript) in a 5% CO_2_ atmosphere at 37°C. After culturing for 1 month, beads were added to cells seeded in 96 wells or to the axon well of nerve organoid cultured in the B-type microchip. These cells or nerve organoid were further cultured for another 48 h to promote synapse formation, after which they were used for testing.

#### Cell culture (HEK293T)

We used the commercial HEK293T cell line (ATCC) that were induced for SYT2 stable expression cell lines and were generated at MBL. Cells were grown in DMEM (Gibco) supplemented with 1% penicillin-streptomycin (Thermo Fisher Scientific), 10% FBS (Gibco), and 1% GlutaMAX (Thermo Fisher Scientific) in a 5% CO_2_ atmosphere at 37°C.

#### Human tissues study

Thirty-micrometer-thick transverse cryosections were cut on a cryostat (Leica), collected onto silanized slides, and stored at −80°C. The sections were fixed with ice-cold acetone for 10 min, and dried. After washing in Tris-buffered saline (TBS), pH 7.4, nonspecific binding was blocked with 5% normal goat serum (Vector) and 0.3% Triton X-100 in TBS for 20 min at room temperature. The sections were then incubated with primary antibody (LRRTM2, Novus Biologicals) and alpha-bungarotoxin, Alexa 594 conjugate (Thermo Fisher Scientific) in the blocking solution for 24 h at 4°C and then rinsed extensively in TBS, followed by incubation with the appropriate secondary antibodies conjugated to fluorochromes (Alexa Fluor 488, Thermo Fisher Scientific) in Antibody Diluent (Dako) overnight at 4°C. Subsequently, the slides were washed extensively in TBS, dipped into distilled water, coverslipped with PermaFluor (Thermo Fisher Scientific), and stored in the dark at 4°C. We analyzed the fluorophore-labeled sections under a confocal laser scanning microscope system with Ar (488 nm), and HeNe-green (594 nm) laser units (IX 71 and FV300; Olympus). Digital images were captured using acquisition software (Fluoview version 4.3; Olympus).

#### Rabbit SYT2 uptake study

Human MN cells were maintained in Neurobasal Plus Medium (Thermo Fisher Scientific), 2% B-27 Plus Supplement (50×) (Thermo Fisher Scientific), 1% penicillin-streptomycin (Thermo Fisher Scientific), 20 ng/mL BDNF (GenScript), and 20 ng/mL GDNF (GenScript). This study used 10 μg/mL SYT2 antibody luminal domain (Synaptic Systems), 10 μg/mL normal rabbit IgG control (R&D Systems), and 10 μg/mL SYT2 antibody cytoplasmic domain (Synaptic Systems). To enhance antibody uptake, a mixture of antibody and 100 μM 4AP (Sigma-Aldrich) was added and incubated at 37°C for 30 min, then washed with culture medium. Finally, cells were fixed with 2% paraformaldehyde (PFA) (NACALAI TESQUE).

#### Immunofluorescence staining (cell)

Human MN cells and nerve organoid were fixed in 2% PFA (NACALAI TESQUE) and then washed in wash buffer (0.02% Triton X-100 [Tx, NACALAI TESQUE] in PBS [WAKO]). Cells were then incubated in permeabilizing buffer (0.25% Tx in PBS) and blocking solution (2% normal goat serum [NGS]; Thermo Fisher Scientific), 1% BSA (NACALAI TESQUE), 1% fetal bovine serum (Gibco), and 0.02% Tx in PBS at room temperature for 1 h with gentle agitation. Detection of targets was performed by incubating cells in a cocktail of primary antibodies for a day at 4°C under agitation. The cell was then washed with wash buffer and incubated with Alexa Fluor-conjugated secondary antibodies (Invitrogen) for 1 h at room temperature under agitation. After additional rinses in wash buffer, cells were treated with 1% PFA for re-crosslinking. Primary antibody cocktails were composed of antibodies from different species or different isotypes of IgG. A list of primary antibodies used in this study and their respective dilutions can be found in the key resources table. Microscope (Leica DMi8), camera (DFC9000GTC), and filter (XLED-Q-P) were used for imaging. ImageJ v1.53e and Meta Morph version 7.10.2.240 were used for analysis.

#### SYT2 monoclonal antibodies selection and generation

Monoclonal SYT2 antibody, selection, generation and production was outsourced to Medical & Biological Laboratories Co. (MBL). The process is illustrated in Figure S1. Briefly, the immunogen and panning antigens were synthesized by Beijing SciLight Biotechnology. The sequences of the synthesized peptides for immunogen and panning are as follows.

Immunogen peptides

Human full-length of SYT2 luminal domain; h2F-Ag (1–62 aa), MRNIFKRNQEPIVAPATTTATMPIGPVDNSTESGGAGESQEDMFAKLKEKLFNEINKIPLPP-C.

Human partial length of SYT2 luminal domain; h2P-Ag (1–25 aa), MRNIFKRNQEPIVAPATTTATMPIG-C.

Panning peptides

Human SYT2 full luminal domain; h2F-bio (1–62 aa), MRNIFKRNQEPIVAPATTTATMPIGPVDNSTESGGAGESQEDMFAKLKEKLFNEINKIPLPP-K-biotin.

Mouse SYT2 full luminal domain; m2F-bio (1–65 aa), MRNIFKRNQEPNVAPATTTATMPLAPVAPADNSTESTGPGESQEDMFAKLKEKFFNEINKIPLPP-K-biotin.

Human SYT1 full luminal domain; h1F-bio (1–58 aa), MVSESHHEALAAPPVTTVATVLPSNATEPASPGEGKEDAFSKLKEKFMNELHKIPLPP-K-biotin.

Keyhole limpet hemocyanin (KLH) was conjugated to the peptide and the concentration was prepared to 1.0 mg/mL. The solution was immunized once a week for a total of four times to two each of Balb/c mice and diseased mice. Total RNA was extracted from lymph node cells of h2F-Ag-KLH and h2P-Ag-KLH immune mice, and cDNA was prepared. VH and VL genes were amplified using primers for antibodies. The VH and VL genes were incorporated into a phagemid vector and transfected in *E. coli* to generate antibody fragment-expressing phage library. SYT2 antigen-specific antibody phages were enriched with three rounds of panning (Table S5). In each of the first two rounds of panning, the phage library was reacted with excluding antigen, human SYT1 full luminal domain, h1F-bio, to remove non-SYT2-specific antibodies. Human SYT2 full luminal domain antigen, h2F-bio, was used in the first round of panning and mouse SYT2 full luminal domain antigen, m2F-bio, was used in the second round of panning. The final round of panning was performed with human SYT2-expressing HEK293T stable cell lines and non-specific antibodies were excluded with HEK293T cells alone. Clones of scFv identified by panning were induced with isopropyl β-D-thiogalactopyranoside (IPTG) and polyclonal single-chain variable fragment (scFv) were obtained from culture supernatants. ELISA of polyclonal scFv was performed to evaluate the enrichment of SYT2 peptides antigen-specific scFv clones. Monoclonal h2F and h2P scFv libraries obtained from polyclonal scFv ELISA were seeded onto plates for cloning. Monoclonal scFv were obtained from IPTG-induced the culture supernatant. Monoclonal scFv ELISA was performed to evaluate the enrichment of SYT2 peptides antigen-specific clones and aa sequences of these enriched scFv clones were analyzed by Azenta US. According to the aa sequence homology, scFV clones were arranged into clusters. SYT2 scFVs were further evaluated for monoclonal enrichment against hSYT2 antigens with human SYT2-expressing HEK293T cells using FCM. Positive SYT2 scFV clones were IPTG induced and the culture supernatant containing scFV was concentrated 10-fold by ammonium sulfate precipitation, dialyzed, and filtered through a 0.22-μm filter. The final selections for SYT2 scFV clones were evaluated for affinity binding and internalization ability at MN pre-synapse with the induced-pre-synapse *in vitro* model. Competent SYT2 scFv clones were isolated, purified, and converted to human-mouse chimera IgG1 antibodies. The antibodies mainly used in this study were FL08, PL13, and PL20.

#### Kinetics measurement

Kinetics measurement was outsourced to MBL. For the antibodies produced, affinity (K_D_), binding kinetics, and dissociation kinetics to biotinylated h2F peptides were measured. Instrument used for the measurements was an Octet RED96e system (Sartorius). The biosensor used was octet streptavidin Biosesor (Sartorius). Concentrations of the measured samples were 5, 2.5, 1.25, 0.63, 0.31, 0.17, 0.08, and 0 nM. Sensitizing antigen was 0.2 μg/mL or 0.05 μg/mL. Buffer contains 0.02% Tween 20/0.01% BSA/PBS.

#### Acetylcholine assay

Two days before pre-synapse induction with LRRTM2 or IgG coated microbeads, nerve organoid were washed and cultured in Hank’s balanced salt solution (HBSS)(+) with calcium and magnesium solution that is without phenol red (Nacalai Tesque) supplemented with 2% B-27 Plus Supplement (50×) (Thermo Fisher Scientific), 1% penicillin-streptomycin (Thermo Fisher Scientific), 20 ng/mL BDNF (GenScript), and 20 ng/mL GDNF (GenScript). Microbeads were added to the axon well of B-type microchip and incubated for 48 h to promote synapse formation. SV release at the pre-synapse were stimulated with HBSS(+) medium containing 100 μM 4AP and 15 μM itopride hydrochloride (Selleck) for 1 h. Supernatant was harvested from the axon well and acetylcholine was evaluated with a commercial kit (Abcam).

#### Antibody distribution (*in vivo*)

The animals used and the breeding conditions were described in the experimental animals section. In this study, PL13 antibody or Human IgG1 isotype control chimeric mAb (MBL) was used, and i.v. administered at a single injection dose of 5 mg/kg. The mice were fixed, and organs were harvested at each time point (12, 24, 72, and 240 h after i.v. injection). Under isoflurane inhalation anesthesia, the mice were perfused with PBS into the left ventricle and euthanized. After confirming that the blood in the body had been drained, PBS was replaced and fixed with 4% PFA. After perfusion, each organ was harvested from the mice, and blood and other material were washed away with PBS, and then each organ was immersed in 4% PFA for 1 day. The tissue was then sucrose-substituted with 10% sucrose (WAKO) for 2 h (4°C), 20% sucrose for 2 h (4°C), and 30% sucrose overnight (4°C), and embedded in OCT compound (Sakura Finetek Japan). Sections were made with a Leica cryostat and were 10 μm thick.

#### Immunofluorescence staining (tissue)

Sections were washed in wash buffer (0.1% Tx [NACALAI TESQUE] in PBS [WAKO]). Sections were incubated in permeabilizing buffer (0.25% Tx in PBS) and then blocking solution (3% NGS [Thermo Fisher Scientific] or 3% normal donkey serum [Merck], 2% BSA [NACALAI TESQUE], 0.1% Tx in PBS) at room temperature for 1 h. Detection of targets was performed by incubating sections in a cocktail of primary antibodies for 1 day at 4°C under agitation. The tissue was then washed with wash buffer and incubated with Dylight or Alexa Fluor-conjugated secondary antibodies (Invitrogen) and or 1.43 μM of DAPI (Invitrogen) for 1 h at room temperature. After additional rinses in wash buffer, sections were mounted with Fluoromount-G (Southern Biotechnology Associates). A microscope (Leica DMi8) and camera (DFC9000GTC) with a filter (XLED-Q-P) was used for imaging. Meta Morph version 7.10.2.240 and ImageJ v1.53e software were used for analyses.

#### RI test

The RI test was outsourced to Nihon Medi-Physics Co. Briefly, the study design involved the use of animals, this was conducted in compliance with the relevant regulations and standards in accordance with the Regulations on Safety Management of Biological Experiments of the Research Facilities of Nihon Medi-Physics Co. A total of 12 mice with normal ICR were obtained from Jackson Laboratory Japan, including one spare mouse per group for a total of three mice per antibody (total of nine mice per group) for this study. Before Zr^89^ labeling, FL08, PL20, and control IgGs were modified with CCAP (Chemical Conjugation by Affinity Peptide). After the Zr^89^-labeling , Zr^89^-labeled FL08, PL20, and control IgG antibodies were administered intravenously to the mice. Evaluation of RI Zr^89^ labeled FL08, PL20, and human IgG1 isotype control chimeric antibody (Control IgG, MBL) was by PET imaging of Zr^89^-labeled FL08, PL20, and control IgG. Organs were harvested after 78–79 h for radioactivity measurement with gamma-ray well scintillation measurement system.

#### MMAE uptake study

Cell culture was performed as described in Cell culture (MN). The conjugation of VcMMAE (mc-vc-PAB-MMAE) (MedChemExpress) to each antibody was performed using MagicLink Protein Protein Crosslinking Kit (BroadPharm) following the manufacturer’s protocol. The conjugation molar ratio of antibody to MMAE is 1:2. The MMAE uptake test was performed as in Rabbit SYT2 uptake test. The final concentration of each reagent was 10 μg/mL. Microscope (Leica DMi8), camera (DFC9000GTC), and filter (XLED-Q-P) were used for imaging. ImageJ v1.53e was used for analysis. Primary and secondary antibodies are listed in Table S4.

#### Antibody oligonucleotide conjugate *in vitro* study

##### Generation of antibody oligonucleotide conjugates

For antibody oligonucleotide conjugate (AOC) generation, nucleic acid synthesis with linkers was outsourced to Ajinomoto Bio-Pharma to synthesize anti *MALAT1* ASO (C6-(5′-) GmCATTmCTAATAGmCAGmC (−3′)) and randomized ASO (C6-(5′-) TmCAmCTmCGAAmCAGTAGT (−3′)). ASO and DBCO-PEG4-Val-Cit-PAB-PNP (BroadPharm) were then conjugated as per protocol using oYo-Link Azide (AlphaThera) and LED PX2 Photo-Crosslinking Device (AlphaThera). The conjugation molar ratio of antibody to the synthesized nucleic acids with linkers was 1:3. Conjugation was confirmed by SDS-PAGE.

##### AOC binding evaluation study

HEK293T cells were seeded at 1 × 10^4^/well on Geltrex hESC-Qualified, Ready-To-Use, Reduced Growth Factor Basement Membrane Matrix (Thermo Fisher Scientific)-coated Black Microplate Flat Bottom 96 Well, I type gamma ray sterilized (AS ONE). The test was performed when the cells were 100% confluent. Each reagent was dissolved in medium and prepared to a final concentration of 1 μg/mL was incubated at 37°C, 5% CO_2_ for 1 h, then washed with medium. The cells were fixed at a final concentration of 2% PFA and then immunofluorescence stained. Fluorescence intensity was measured at GloMax Discover Microplate Reader (Promega).

##### AOC RNA knockdown study

For the HEK293T experiment, cells were seeded at 4 × 10^5^/well on Cell Culture Multiwell plate, six-well, PS, clear (Greiner). The test was performed when the cells were approximately 70% confluent. Randomized ASOs and *MALAT1* ASOs were prepared to a final concentration of 10 μM. DBCO Linker conjugate *MALAT1* ASO was prepared at 0.1, 1, and 10 μM. The incubation conditions were 37°C, 5% CO_2_ for 2 days. The MN experiment, cell culture was performed as described in Cell culture (MN). This study was performed as described in rabbit SYT2 uptake test. Conjugated PL13-*MALAT1*-ASOs or *MALAT1*-ASOs alone were added to the cells at final concentrations of 10 and 2 μg/mL and incubated at 37°C for 2 days. In each experiment, cells were washed with PBS (WAKO) after incubation and collected in 0.125% trypsin solution (NACALAI TESQUE). RNA extraction, cDNA synthesis, and qPCR are described in quantitative PCR for RNA expression.

#### AOC *in vivo* study

##### Animal study

This test was outsourced to KAC Co. The animals used and the breeding conditions were described in the experimental animals section. Briefly, this study was based on work by Beaudet et al. (Sci Rep 2015)^45^. In this study, C57Bl/6 mice purchased from Jackson Laboratories were used. Each group had 3 mice and a total of 18 mice (*n* = 3). PL13-*MALAT1*-ASO conjugated or PL13-randomized-ASO conjugated was used, and i.v. injected at a dose of 5 mg/kg; for FACS, PL13 antibody was administered i.v. at 5 mg/kg 72 h before sampling. Spinal cord cells were isolated using the Papain Dissociation System (Worthington Biochemical Corporation) according to the protocol. The isolated spinal cord cells were fixed with 2% PFA.

#### FACS

FACS was based on Martin et al.^46^ Isolated cells were washed with PBS (WAKO), and cell counts were performed using Countess 3 (Thermo Fisher Scientific). Antibody solutions were prepared by diluting Goat anti-Human IgG (H + L) Cross-Adsorbed Secondary Antibody, Alexa Fluor Plus 488 (20 μg/mL, Invitrogen) and DAPI (1.43 μM, Invitrogen) with staining buffer (1mM DTT [NACALAI TESQUE], 0.1U/μL RiboLock RNase Inhibitor [Thermo Fisher Scientific], and 0.1% Tween [NACALAI TESQUE] in PBS [WAKO]). The cell concentration was adjusted with antibody solution to 1 × 10^5^/50 μL. The adjusted antibody solution was inverted and mixed at 4°C for 30 min in a light-shielded environment. Stained cells were washed with wash buffer (0.1% Tween in PBS). Finally, the cells were diluted with staining buffer to a cell concentration of 1 × 10^7^/mL and used. To identify *Malat1* expression, stained spinal cord cell suspensions were sorted for PL13 and DAPI-positive cells using SORPAria or Aria 3 cell sorter (BD). The analysis software used was FlowJo (BD).

#### Quantitative PCR for RNA expression

For qPCR analysis, total RNA was extracted using the NucleoSpin totalRNA FFPE XS (MACHEREY-NAGEL) or RNeasy Plus Mini Kit (Qiagen), and reverse transcribed with the QuantAccuracy, RT-RamDA cDNA Synthesis Kit (TOYOBO). Gene expression was analyzed by qPCR with TB Green Premix Ex Taq (Tli RNaseH Plus) (Takara Bio). The PCR primers used in this study are listed in Table S4.

### Quantification and statistical analyses

Analyses were performed using Prism 9.0 (GraphPad), and graphs were generated using Prism 9.0. Data represents mean ± SEM. Specific tests (e.g., t test, one-way ANOVA) and significance are indicated in figure legends.

For reagents and resources, refer to Table S4.

## Data and code availability

Data are available upon request. No original code was reported in this paper. Any additional information required to reanalyze the data reported in this paper is available upon request. Materials availability of unique reagents generated in this study are available from Jiksak Bioengineering through a completed materials transfer agreement. Material requests should be sent to info@jiksak.co.jp.

## Acknowledgments

Flow cytometry was performed in the IMSUT FACS Core laboratory. We acknowledge the IMSUT FACS Core laboratory for assistance with flow cytometry analysis. We thank Mami Okamoto, Sanae Ishizuka, Tomomi Sakamoto, Rumiko Izumi, Masako Suzuki, Mai Kakinuma, and Hinako Shigihara for general technical support and Tetsuya Akiyama and Prof. Masashi Aoki for useful discussions (Tohoku University, Japan). Funding for this work was provided by Jiksak Bioengineering. Graphic abstract was created with BioRender.com., Yumoto, N. (2025) https://BioRender.com/c67b531.

## Author contributions

The paper was conceptualization by N.Y. The methodology was designed by K.K.L.Y., J.K., D.I., and N.Y. The data were obtained by K.K.L.Y., J.K., R.H., and D.I. Formal analysis of the data was performed by K.K.L.Y., J.K., and D.I. The manuscript was drafted by K.K.L.Y., J.K., and N.Y. K.K.L.Y., J.K., D.I., N.S., and N.Y. review and edited the manuscript. N.Y. supervised the work.

## Declaration of interests

N.Y. is a full-time employee, shareholder, and stakeholder in Jiksak Bioengineering. K.K.L.Y., J.K., and D.I. are full-time employees of Jiksak Bioengineering, and hold stock options in the company. This work is described in PCT application number PCT/JP2023/016125 (published as WO2023210585A1), entitled “Targeting Agent,” with N.Y. and D.I. as authors. mAb-SYT2, FL08, and PL20 are described in PCT/JP2024/019802 and PCT/JP2024/019803, entitled “SYT2 Antibody,” with N.Y. as an author. Additional mAb-SYT2 patents have been submitted to the Japanese Patent Office under application numbers 2023-180229, 2023-180235, 2023-180242, 2023-180358, 2023-180359, and 2023-180361, are all entitled “SYT2 Antibody,” with N.Y. as an author.

The authors declare that these financial relationships do not influence the objectivity of the research presented.

## Declaration of generative AI and AI-assisted technologies in the writing process

During the preparation of this work, the author(s) used ChatGPT and Claude 3.5 Sonnet to improve on readability. After using this tool or service, the author(s) reviewed and edited the content as needed and take(s) full responsibility for the content of the publication.

## Supplemental information

Document S1. Figures S1–S8, Tables S1–S3 and S5

Table S4. Reagents and resources

Document S2. Article plus supplemental information
