# Supplementary material for: Harnessing synaptic vesicle release and recycling with antibody shuttle for targeted delivery of therapeutics to neurons
Source: Mol Ther Methods Clin Dev. 2025 Apr 19;33(2):101476. doi: 10.1016/j.omtm.2025.101476 (PMC12124621; doi:10.1016/j.omtm.2025.101476)
Supplement: Document S1. Figures S1–S8, Tables S1–S3 and S5 [file mmc1.pdf]

OMTM, Volume *33*

Supplemental information

Harnessing synaptic vesicle release and recycling with antibody shuttle for targeted delivery of therapeutics to neurons

Karen Kar Lye Yee, Junichi Kumamoto, Daijiro Inomata, Naoki Suzuki, Ryuhei Harada, and Norihiro Yumoto

Table S1. Sequences of FL and PL selected scFV clones

FL03	
H-chain	QVQLQQSGAELVRPGASVKLSCKASGYTFTDYEMHWVKQTPVHGLEWIGAIDPETGGTAYNQKFKGK ATLTADKSSSTAYMELRSLTSEDSAVYYCTRGN--DGYN-E-DYWGQGTSLTVSS
H-chain	QVQLQQSGAELVRPGASVKLSCKASGYTFTDYEMHWVKQTPVHGLEWIGAIDPETGGTAYNQKFKGK ATLTADKSSSTAYMELRSLTSEDSAVYYCTRGN--DGYN-E-DYWGQGTSLTVSS
FR1	QVQLQQSGAELVRPGASVKLSCKASGYTFT
CDR1	DYEMH
FR2	WVKQTPVHGLEWIG
CDR2	AIDPETGGTAYNQKFKG
FR3	KATLTADKSSSTAYMELRSLTSEDSAVYYCTR
CDR3	GN--DGYN-E-DY
FR4	WGQGTSLTVSS
L-chain	DIVMTQSPLTLSVTIGQPASISCKSSQSLLDSDGKTYLNWLLQRPGQSPKRLIYLVSKLDSGVPDRFTGS GSGTDFTLKISRVEAEDLGVYYCWQDTHFPYTFGGGTKLEIK
FR1	DIVMTQSPLTLSVTIGQPASISC
CDR1	KSSQSLLDSDGKTYLN
FR2	WLLQRPGQSPKRLIY
CDR2	LVSKLDS
FR3	GVPDRFTGSGSGTDFTLKISRVEAEDLGVYYC
CDR3	WQDTHFPYT
FR4	FGGGTKLEIK
FL08	
H-chain	EVQLQESGAELVRPGASVKLSCKASGYTFTDYEMHWVKQTPVHGLEWIGAIDPETGGTAYNQKFKGKA TLTADKSSSTAYMELRSLTSEDSAVYYCTRGN--DGYN-E-DYWGQGTTLTVSS
FR1	EVQLQESGAELVRPGASVKLSCKASGYTFT
CDR1	DYEMH
FR2	WVKQTPVHGLEWIG
CDR2	AIDPETGGTAYNQKFKG
FR3	KATLTADKSSSTAYMELRSLTSEDSAVYYCTR
CDR3	GN--DGYN-E-DY
FR4	WGQGTTLTVSS
L-chain	DVLMTQTPLTLSVTIGQPASISCKSSQSLLDSDGKTYLNWLLQRPGQSPKRLIYLVSKLDSGVPDRFTGS GSGTDFTLKISRVEAEDLGVYYCWQDTHFPYTFGGGTKLEIK
FR1	DVLMTQTPLTLSVTIGQPASISC
CDR1	KSSQSLLDSDGKTYLN
FR2	WLLQRPGQSPKRLIY
CDR2	LVSKLDS
FR3	GVPDRFTGSGSGTDFTLKISRVEAEDLGVYYC
CDR3	WQDTHFPYT
FR4	FGGGTKLEIK
PL09	
H-chain	QVQLQQSGAEFVRPGASVKLSCTTSGFNI-KDDYM-HWVKQRPEQGLEWIGRIDPEN GDTEFASKFQGKATITADTSSNTAYLQLSSLTSEDTAVYYCSTDYYTHPFA---YWGQGTLVTVSA
FR1	QVQLQQSGAEFVRPGASVKLSCTTSGFNI-
CDR1	KDDYM-H
FR2	WVKQRPEQGLEWIG
CDR2	RIDPENGDTEFASKFQG
FR3	KATITADTSSNTAYLQLSSLTSEDTAVYYCST
CDR3	DYYTHPFA---Y
FR4	WGQGTLVTVSA
L-chain	DIVMTQAAPSVPVTPGESVSISCRSSKSLL-HSDGNTYLYWFLQRPGQSPQLLIYRMSNLASGVPDRF SGSGSGTAFTLRISGVEAEDVGVYFCLQRLEYPFTFGSG-TKLEIK
FR1	DIVMTQAAPSVPVTPGESVSISC
CDR1	RSSKSLL-HSDGNTYLY
FR2	WFLQRPGQSPQLLIY
CDR2	RMSNLAS
FR3	GVPDRFSGSGSGTAFTLRISGVEAEDVGVYFC
CDR3	LQRLEYPFT
FR4	FGSG-TKLEIK
PL10	

H-chain	EVQLQQSGAELVRPGASVKLSCTTSGFNI-KDDYM-HWVKQRPEQGLEWIGWIDPENGDTGYASKF QGKATITADTSSNTAYLQLGSLTSEDTAVYYCTTDYYNYPFA---YWGQGTLVTVSA
FR1	EVQLQQSGAELVRPGASVKLSCTTSGFNI-
CDR1	KDDYM-H
FR2	WVKQRPEQGLEWIG
CDR2	WIDPENGDTGYASKFQG
FR3	KATITADTSSNTAYLQLGSLTSEDTAVYYCTT
CDR3	DYYNYPFA---Y
FR4	WGQGTLVTVSA
L-chain	DIVMTQAAPSVPVTPGESVSISCRSSKSLL-HSDGNTYLYWFLQRPGQSPQLLIYRMSNLASGVP DRFSGSGVRNCFHTENQWSGGECGCLFLVATARNIHSRSARG-QSWK-N
FR1	DIVMTQAAPSVPVTPGESVSISC
CDR1	RSSKSLL-HSDGNTYLY
FR2	WFLQRPGQSPQLLIY
CDR2	RMSNLAS
FR3	GVPDRFSGSGVRNCFHTENQWSGGEC
CDR3	GCLFLVATARNIHSR
FR4	SARG-QSWK-N
PL13	
H-chain	EVQLQQSGAELVRPGASVKLSCTASGFNI-KDDYV-HWVKQRPEQGLEWIGWIDPENGDTEYAS KFQGKATITADASSNAAYLQLSSLTSEDTAAYYCTTDYYNNPFA---YWGQGTLVTVSA
FR1	EVQLQQSGAELVRPGASVKLSCTASGFNI-
CDR1	KDDYV-H
FR2	WVKQRPEQGLEWIG
CDR2	WIDPENGDTEYASKFQG
FR3	KATITADASSNAAYLQLSSLTSEDTAAYYCTT
CDR3	DYYNNPFA---Y
FR4	WGQGTLVTVSA
L-chain	QIVLTQSPAIMSASPGEKVTITCSASSSV------S-YMHWFQQKPGTSPKLWIYSTSKLASG VPVRFSGSGSGTSYSLTISRMEAEDAATYYCQQRSSYPFTFGSG-TKLEIK
FR1	QIVLTQSPAIMSASPGEKVTITC
CDR1	SASSSV------S-YMH
FR2	WFQQKPGTSPKLWIY
CDR2	STSKLAS
FR3	GVPVRFSGSGSGTSYSLTISRMEAEDAATYYC
CDR3	QQRSSYPFT
FR4	FGSG-TKLEIK
PL20	
H-chain	QVQLKQSGAELVRPGASVKLSCKASGYTF-TDYEM-HWVKQTPVHGLEWIGALDPGTGDTAY KQKFKGKATLTADKSSSTAYMVLRSLTSEDSAVYYCTRGASYSNYE--DYWGQGTSLTVSS
FR1	QVQLKQSGAELVRPGASVKLSCKASGYTF-
CDR1	TDYEM-H
FR2	WVKQTPVHGLEWIG
CDR2	ALDPGTGDTAYKQKFKG
FR3	KATLTADKSSSTAYMVLRSLTSEDSAVYYCTR
CDR3	GASYSNYE--DY
FR4	WGQGTSLTVSS
L-chain	DVVMTQTPLTLSVTIGQPASISCKSSQSLL-DSDGKTYLNWLLQRPGQSPKRLIYLVSKLD SGVPDRFTGSGSGTDITLRISTETQKMEFIIAGIILIFRTRSEGGPSWKDGLM
FR1	DVVMTQTPLTLSVTIGQPASISC
CDR1	KSSQSLL-DSDGKTYLN
FR2	WLLQRPGQSPKRLIY
CDR2	LVSKLDS
FR3	GVPDRFTGSGSGTDITLRISTETQKME
CDR3	FIIAGIILI
FR4	FRTRSEGGPSWKDGLM

Table S2. Dissociation constant and affinity characteristics of selected scFV that were constructed into chimeric full IgG with human Fc

Constructed chimera antibodies were evaluated for their K_D, K_{ON} and K_{OFF} properties. PL13 was characterized with moderate K_D ratio and as a SVRM molecular shuttle this characteristic will allow sufficient SYT2 binding and its release into

the MN.

Peptide	Antibody	K_D	K_{ON}	K_{OFF}	ratio of individual mAb-SYT2 K_D /mAb-SYT2-FL1 K_D
with 0.2 μg/mL SYT2	FL01	1.38E-10	1.56E+05	2.16E-05	1
	FL03	2.75E-10	4.92E+04	1.35E-05	1.99
	PL09	2.74E-10	2.09E+05	5.72E-05	1.99
	PL13	2.47E-10	1.18E+05	2.93E-05	1.79
	PL10	3.16E-10	1.60E+05	5.07E-05	2.29
Peptide	Antibody	K_D	K_{ON}	K_{OFF}	ratio of individual mAb-SYT2 K_D /mAb-SYT2-FL1 K_D
with 0.05 μg/mL SYT2	FL01	1.13E-10	3.78E+05	4.26E-05	1
	FL08	2.67E-11	2.63E+05	7.01E-06	0.24
	PL20	4.48E-11	2.52E+05	1.13E-05	0.40

Table S3. The localization ratio and immunospecificity index of mAb-SYT2 conjugated with Zr^{89}.
The spinal cord showed the highest immunospecificity index for FL08 and PL20. This indicates that mAb-SYT2 is specific and is efficient for spinal cord targeting.

Control IgG	%ID/g	Organ/Blood	ISI
blood	10.30±1.55	1	1
heart	3.53±0.12	0.35±0.05	1
lung	5.43±0.95	0.53±0.02	1
spleen	6.69±0.11	0.66±0.10	1
pancreas	1.48±0.11	0.14±0.01	1
stomach	1.05±0.25	0.10±0.03	1
small intestine	1.82±0.13	0.18±0.02	1
large bowel	1.20±0.02	0.12±0.01	1
testicle	1.99±0.19	0.19±0.02	1
muscle (triceps)	1.44±0.29	0.14±0.03	1
bone (thigh)	3.74±0.21	0.37±0.07	1
kidney	3.01±0.22	0.30±0.06	1
liver	3.99±0.12	0.39±0.04	1
spinal cord	0.50±0.13	0.05±0.01	1
whole brain	0.33±0.06	0.03	1
remaining whole (body)	1.98±0.05	0.19±0.02	1
FL08	%ID/g	Organ/Blood	ISI
blood	4.94±0.99	1	1
heart	2.03±0.12	0.42±0.07	1.22±0.20
lung	4.65±0.42	0.96±0.12	1.82±0.28
spleen	3.68±0.35	0.76±0.11	1.18±0.27
pancreas	0.96±0.10	0.20±0.04	1.38±0.23
stomach	0.47±0.09	0.10±0.01	0.98±0.28
small intestine	0.91±0.17	0.18±0.01	1.05±0.16
large bowel	0.74±0.12	0.15±0.02	1.28±0.09
testicle	1.60±0.15	0.33±0.04	1.70±0.28
muscle (triceps)	1.00±0.18	0.20±0.01	1.49±0.30
bone (thigh)	2.60±0.38	0.53±0.04	1.46±0.24
kidney	2.03±0.14	0.42±0.08	1.47±0.43
liver	2.08±0.36	0.43±0.08	1.11±0.30
spinal cord	0.72±0.08	0.15±0.01	3.20±1.01
whole brain	0.18±0.05	0.04	1.13±0.17
remaining whole (body)	1.05±0.12	0.22±0.02	1.12±0.15
PL20	%ID/g	Organ/Blood	ISI
blood	5.85±1.86	1	1
heart	2.25±0.76	0.38±0.04	1.12±0.18
lung	3.82±1.40	0.65±0.09	1.24±0.21
spleen	4.69±1.79	0.87±0.42	1.41±0.84
pancreas	0.89±0.31	0.15±0.02	1.06±0.17
stomach	0.59±0.18	0.10±0.01	1.06±0.46
small intestine	1.21±0.22	0.21±0.04	1.22±0.34
large bowel	0.81±0.26	0.14±0.01	1.19±0.16

testicle	1.31±0.34	0.23±0.03	1.19±0.24
muscle (triceps)	0.88±0.31	0.15±0.02	1.10±0.19
bone (thigh)	2.76±0.21	0.50±0.15	1.46±0.76
kidney	1.88±0.63	0.32±0.05	1.13±0.33
liver	3.30±0.11	0.61±0.23	1.63±0.82
spinal cord	0.76±0.21	0.13±0.01	2.79±0.67
whole brain	0.24±0.09	0.04±0.01	1.30±0.35
remaining whole (body)	1.12±0.31	0.19±0.02	1.01±0.20

Table S4. Reagents and resources

Excel file provided

Table S5. Reagents and resources used for selection of monoclonal scFv

Target and excluded antigens for each panning round		
Panning Round	Target antigen	Excluded antigen
1st	h2F peptide	h1F peptide
2nd	m2F peptide	h1F peptide
3rd	HEK293T expressing hSYT2	HEK293T
Reagents used for ELISA measurement of polyclonal scFv		
Plate	Immobilizer Streptavidin Plate (Thermo Fisher Scientific, 436020)	
Sensitizing antigen	Biotinylated peptide (biotin-h2F and m2F, h1F 0.1 μg/mL)	
Primary antibody	polyclonal scFv culture supernatant (1x)	
Secondary antibody	Anti-cp3 Rabbit pAbs, (MBL custom product, 5.0 μg/mL)	
Tertiary antibody	Anti-Rabbit IgG Antibody-HRP (MBL #458, 4000-fold dilution)	
Positive control	Synaptotagmin 2 Antibody luminal domain (SYSY #105 223, 5.0μg/mL /mL)	
Negative control	Negative control scFv culture supernatant (1x)	
Substrate	TMB-US (Moss #TMB-US)	
Stop liquid	H2PO4 (FUJIFILM Wako Pure Chemical #167-02166, 0.5 M)	
Reagents used for ELISA measurement of monoclonal scFv		
Plate	Immobilizer Streptavidin Plate (Thermo Fisher Scientific, 436020)	
Sensitizing antigen	Biotinylated peptide (biotin-h2F and m2F, h1F 0.1 μg/mL)	
Primary antibody	monoclonal scFv culture supernatant (1x)	
Secondary antibody	Anti-cp3 Rabbit pAbs, (MBL custom product, 5.0 μg/mL)	
Tertiary antibody	Anti-Rabbit IgG Antibody-HRP (MBL #458, 4000-fold dilution)	
Positive control	Synaptotagmin 2 Antibody luminal domain (SYSY #105 223, 5.0μg/mL /mL)	
Negative control	Negative control scFv culture supernatant (1x)	
Substrate	TMB-US (Moss #TMB-US)	
Stop liquid	H2PO4 (FUJIFILM Wako Pure Chemical #167-02166, 0.5 M)	
Reagents used for FCM measurement of monoclonal scFv		
Device	CytoFLEX (Beckman coulter)	
Cells	SYT2 transgenic HEK293T and HEK293T	
Primary antibody	Monoclonal scFv Culture supernatant (10x)	
Secondary antibody	Anti-cp3 Rabbit pAbs, (MBL custom product, 5.0μg /mL)	
Tertiary antibody	Anti-Rabbit IgG(H+L)-PE (Beckman coulter #IM0855, 200 times diluted)	
Positive control	Synaptotagmin 2 Antibody luminal domain (SYSY #105 223, 5.0μg /mL)	
Negative control	Negative control scFv culture supernatant (1x)	

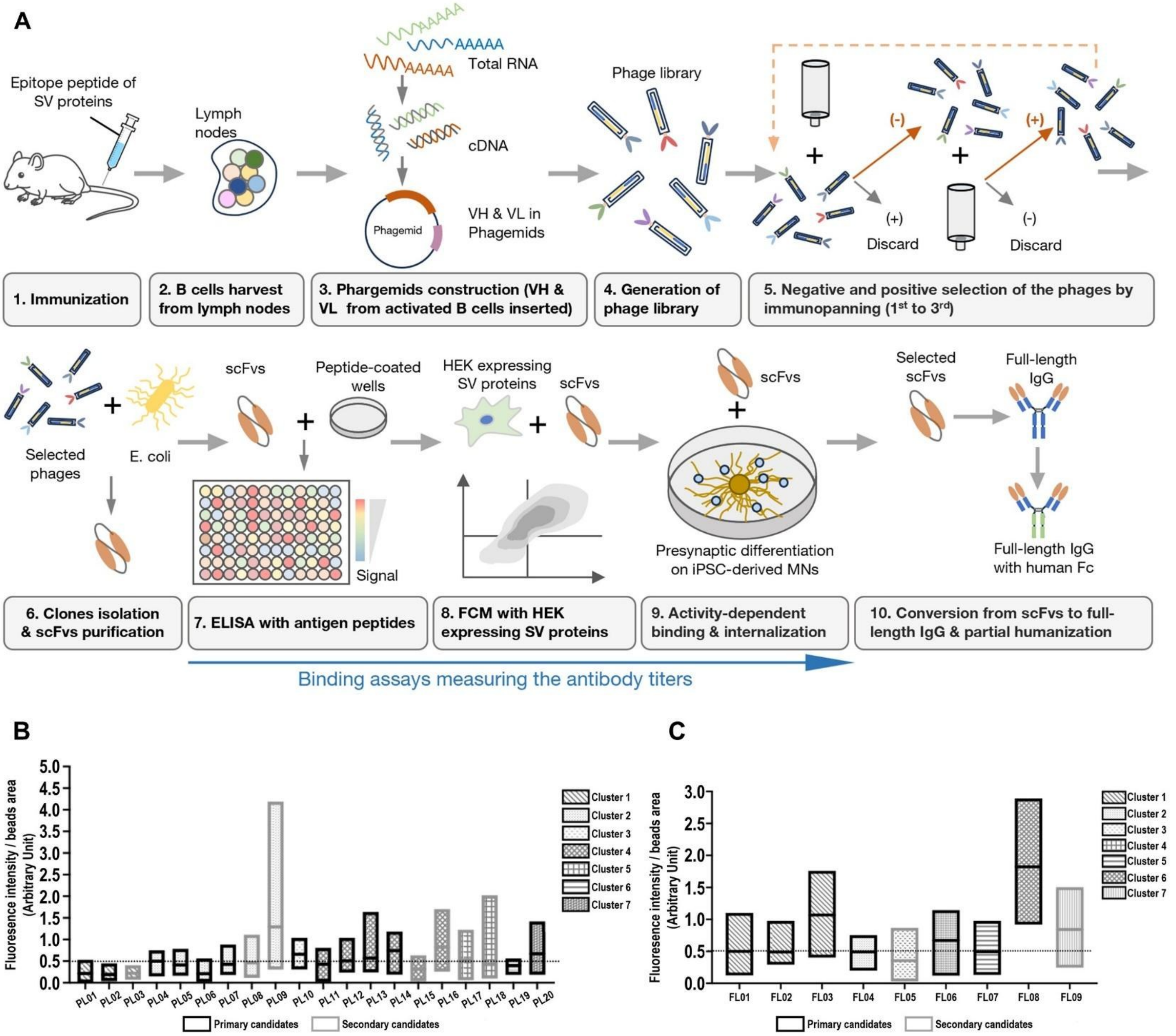

Figure S1. SVRM for molecular shuttling

(A) Illustration for SVRM antibody shuttle screening, identification and generation of monoclonal antibodies specific for human and mouse SV luminal domain proteins. (B-C) Identification of scFVs that binds to SV proteins and were internalized into SV during SVRM stimulation with 4-AP in the pre-synapse induced in vitro model. Graphs shows minimum and maximum value with mean value line. The median value of all the means (0.5) was drawn as a dotted line across the graphs. Further elimination of scFVs below the median of the mean values were done. (B) scFV against partial length SYT2 from AA1-25. Clones above the 0.5 medium value with high, moderate and low internalization into presynapse in the *in vitro* model were selected. PL09 (high), PL10, PL20 (moderate) and PL13 (low) were selected for further study. (C)scFV against full length SYT2 from AA1-62. FL03 and FL08, which have the highest internalization index to presynapse in the *in vitro* model were selected for further study. Data were expressed in mean with minimum and maximum value, n>4.

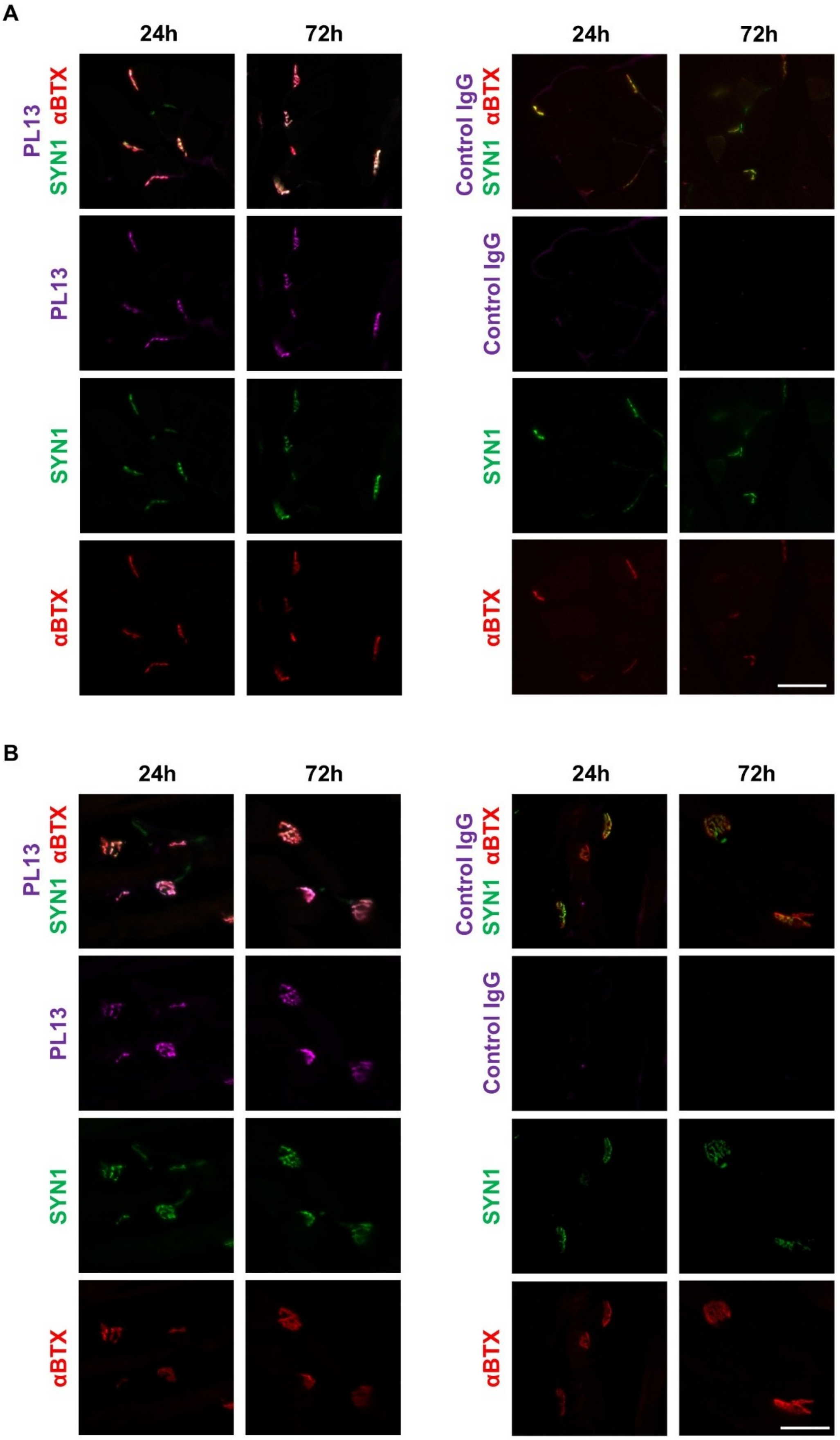

Figure S2. Gastrocnemius and Tibialis Anterior distribution of SVRM molecular shuttles after intravenous administration
(A) PL13 and control IgG (magenta) uptakes into Gastrocnemius after 24h and 72h. Scale bar represents 50µm. (B) PL13 and control IgG (magenta) uptakes into Tibialis Anterior after 24h and 72h. Scale bar represents 50µm.

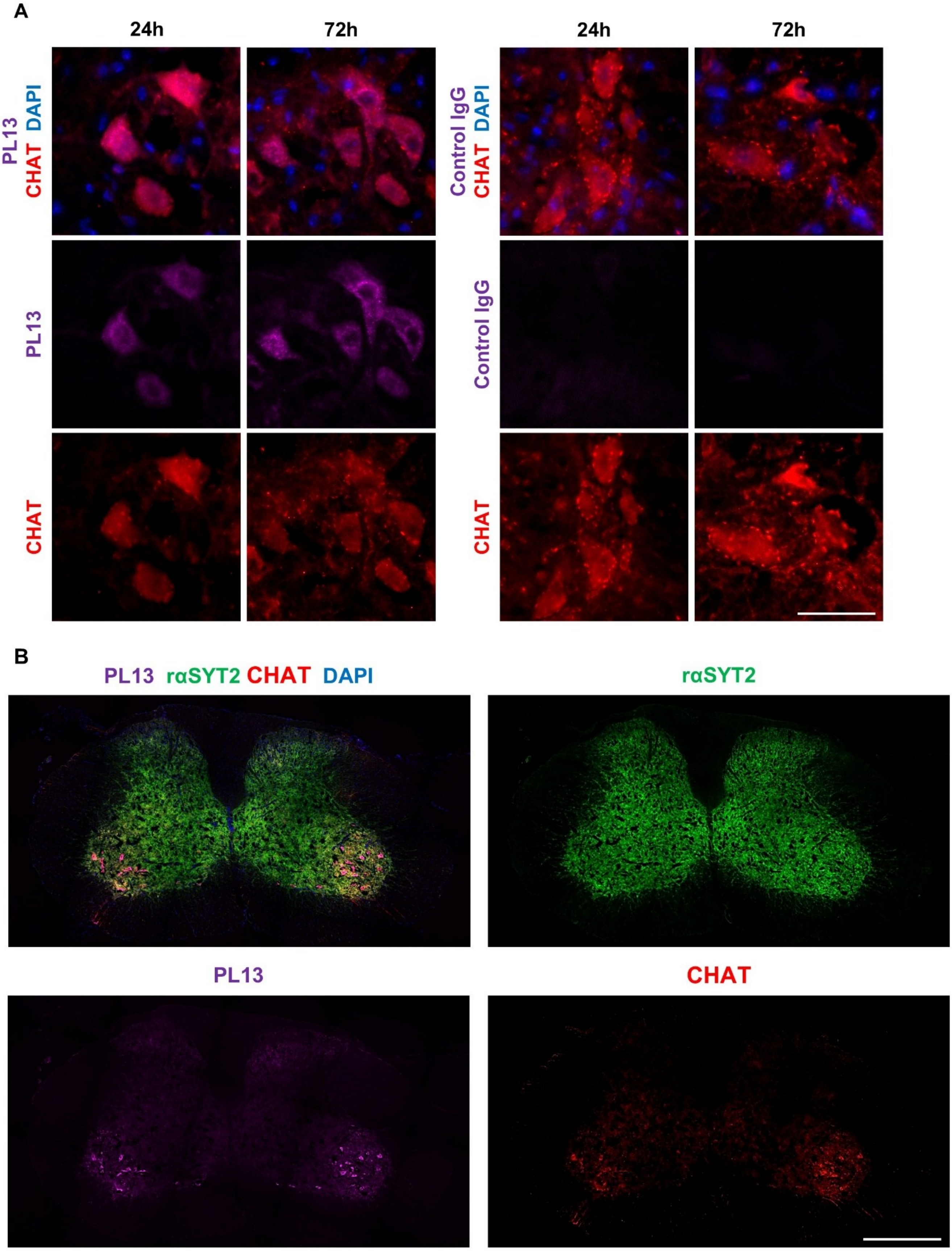

Figure S3. PL13 distribution at the spinal cord
(A) PL13 and control IgG (magenta) uptakes into lumbar motor neuron in the spinal cord after 24h and 72h. Scale bar represents 50μm. (B) Macro image of the spinal cord; PL13 and CHAT stained motor neurons partially co-localization with intrinsic expressing SYT2 at the ventral horn. Images were harvested from mice after 72h intravenous injection of antibody. Scale bar represents 500μm.

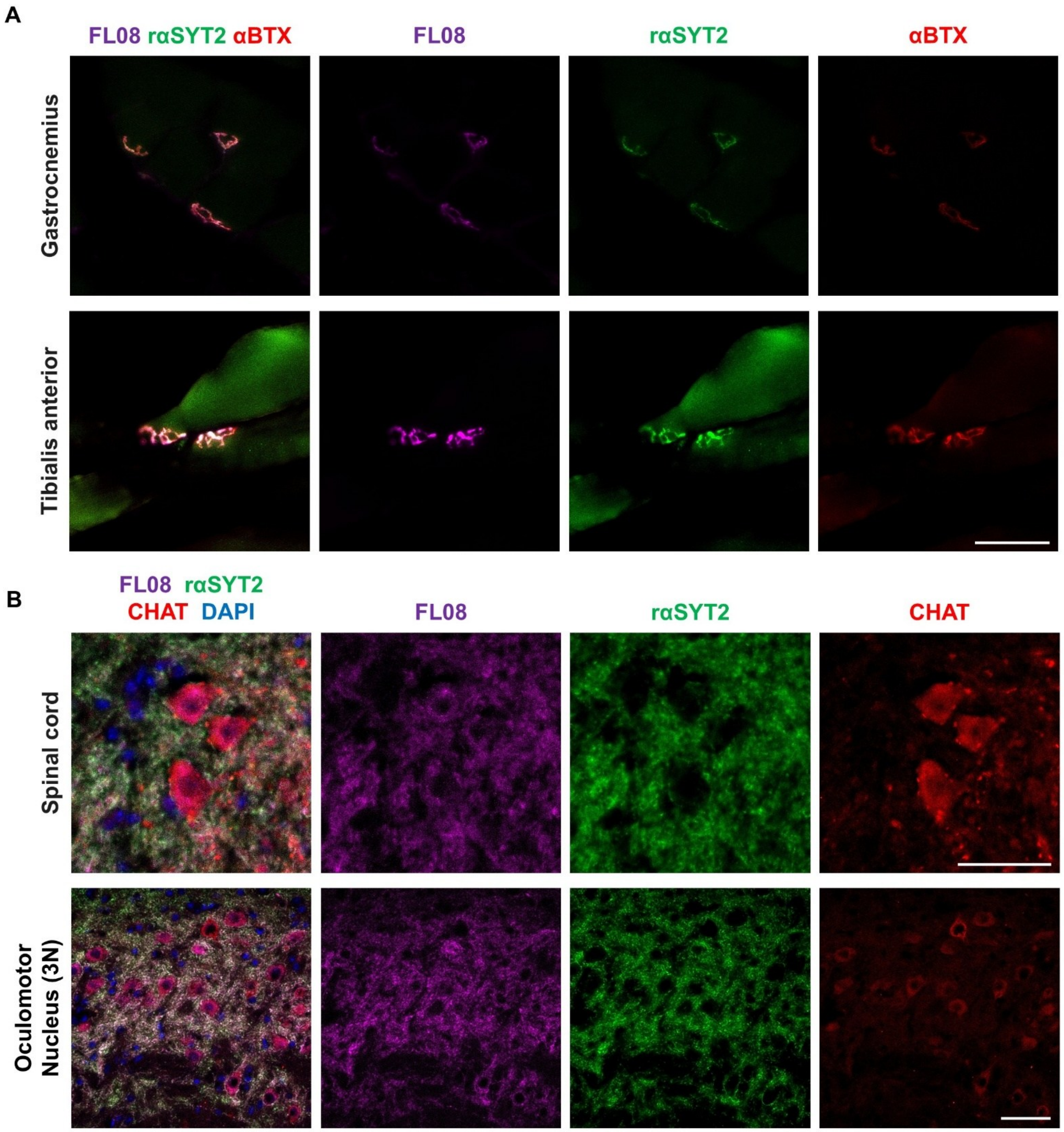

Figure S4. FL08 distribution at NMJ, spinal cord and 3N
(A) FL08 shows uptake by co-localization staining at the NMJ and intrinsic SYT2. (B) FL08 distribution in the spinal cord and brainstem (3N). All images were obtained from mouse tissue after 240h intravenous injection of the antibodies. Scale bar represents 50μm.

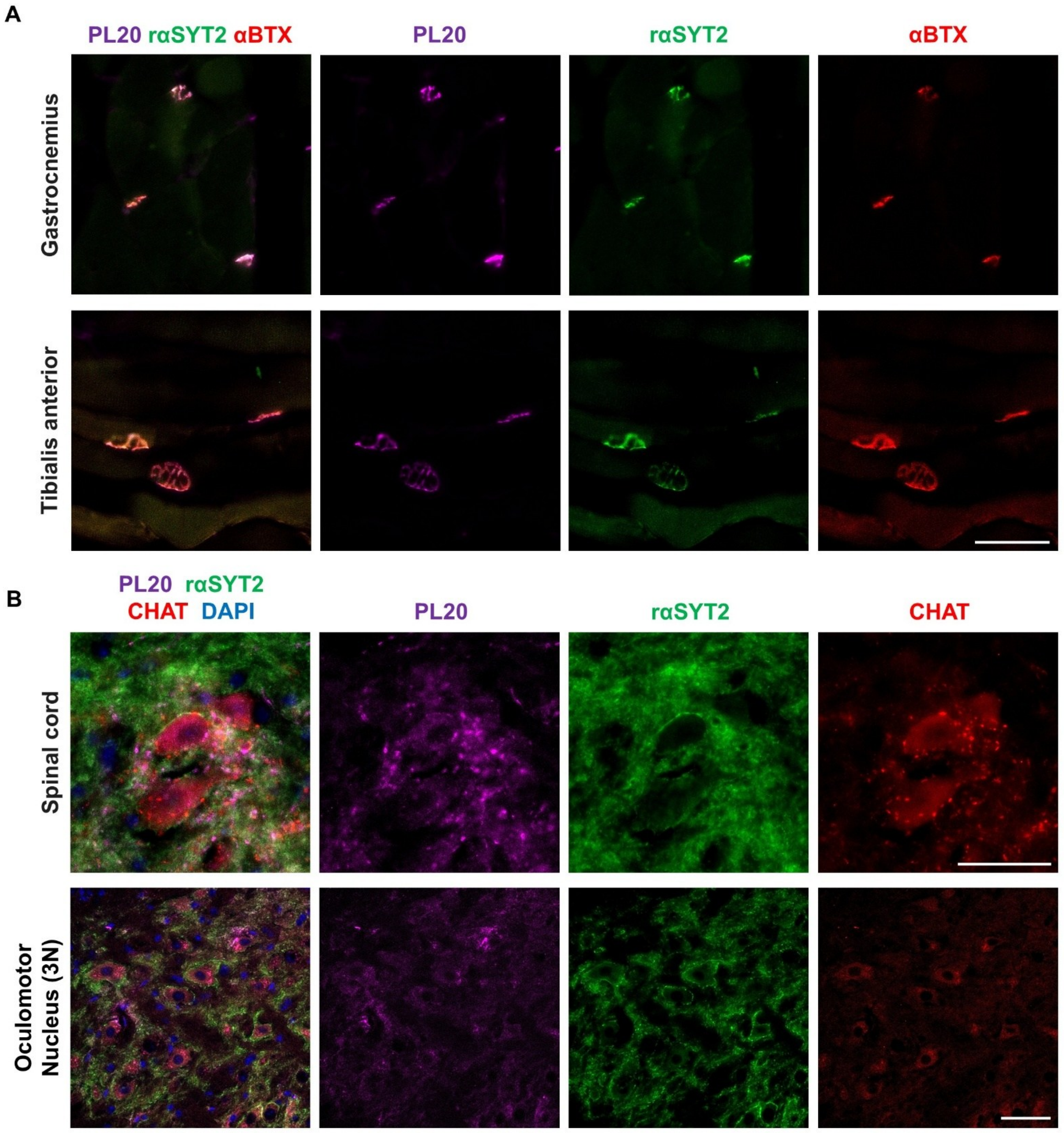

Figure S5. PL20 distribution at NMJ, spinal cord and N3
(A) PL20 shows uptake by co-localization staining at the NMJ and intrinsic SYT2. (B) PL20 distribution in the spinal cord and brainstem (3N). All images were obtained from mouse tissue after 240h intravenous injection of the antibodies. Scale bar represents 50μm.

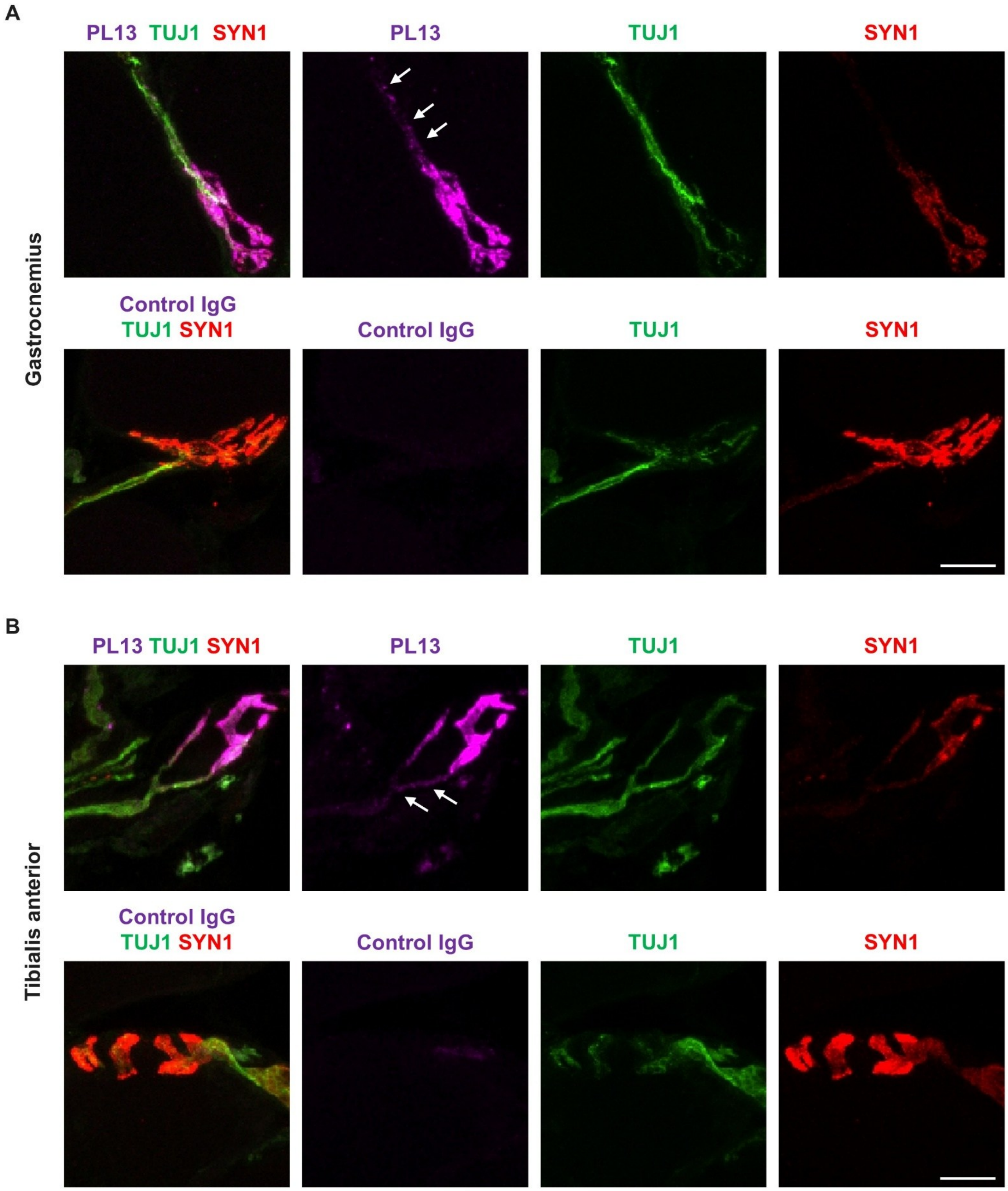

Figure S6. PL13 retrograde from NMJ into axon compared to control IgG
(A) Gastrocnemius (B) Tibialis anterior were harvested from mice after 240h intravenous injection of PL13. Scale bar represents 10μm.

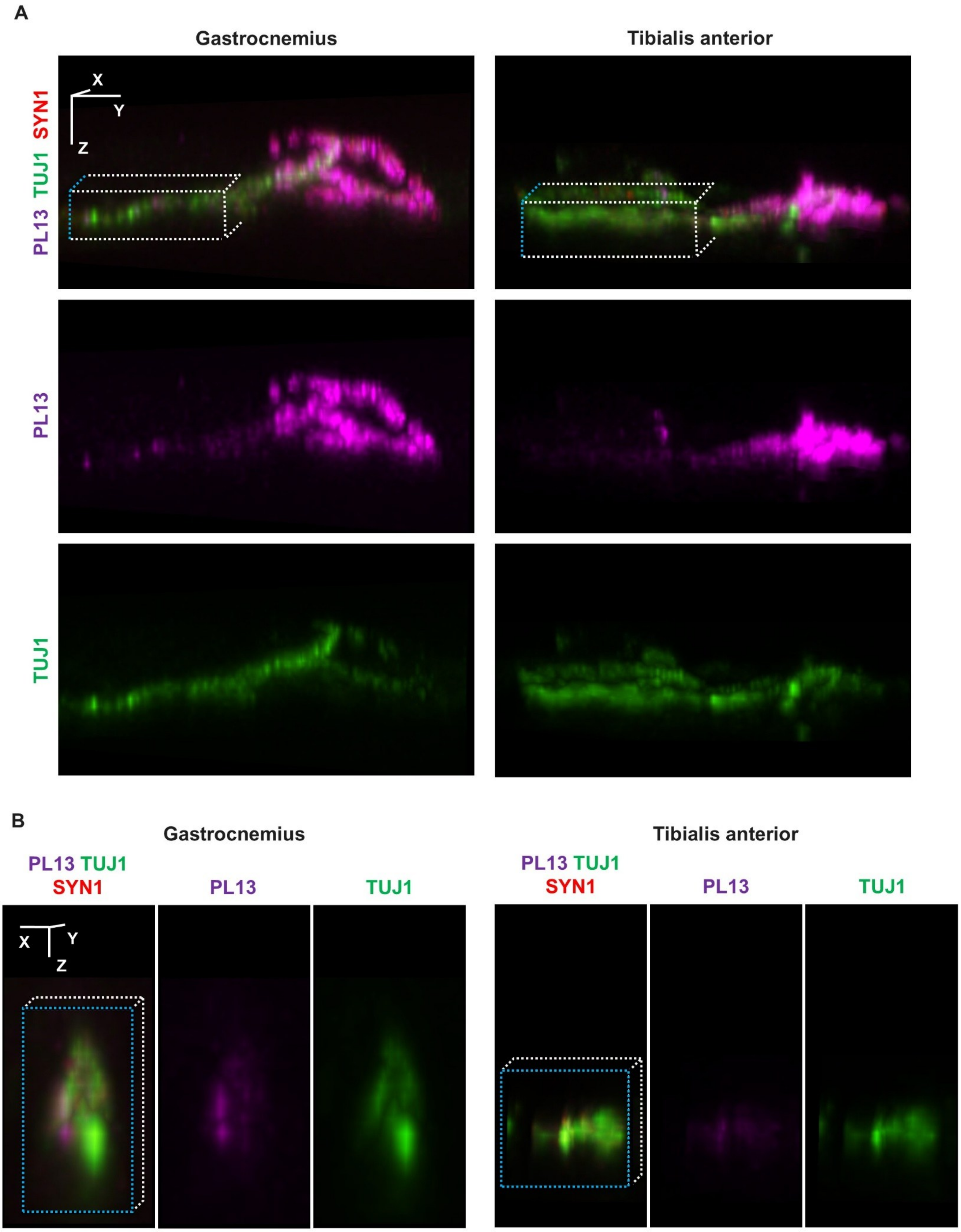

Figure S7. 3D images of PL13 retrogradation from NMJ into axon
(A) Sagittal and (B) Transverse section of the NMJ. Tissues were harvested from mice after 240h intravenous injection of PL13.

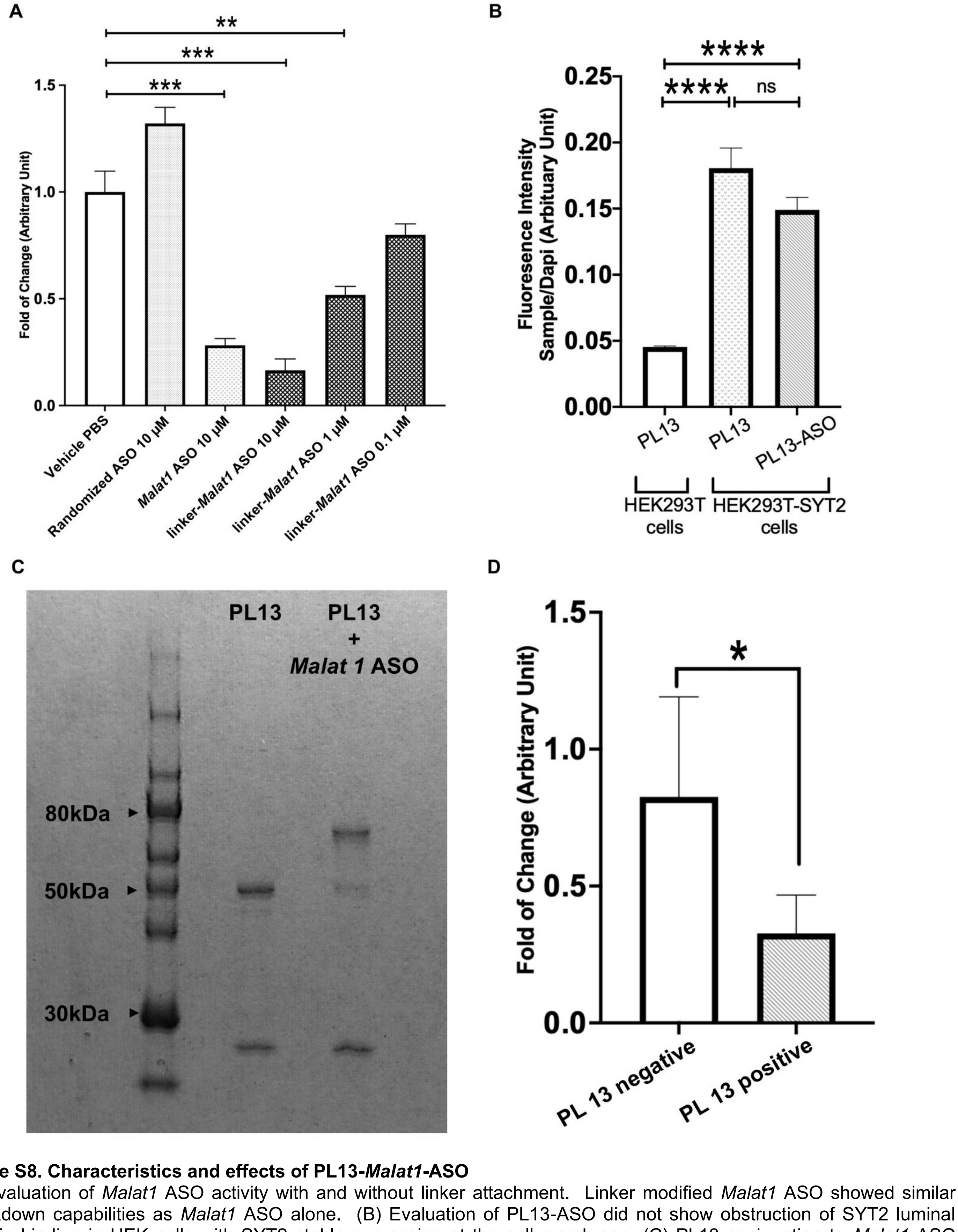

Figure S8. Characteristics and effects of PL13-*Malat1*-ASO

(A) Evaluation of *Malat1* ASO activity with and without linker attachment. Linker modified *Malat1* ASO showed similar knockdown capabilities as *Malat1* ASO alone. (B) Evaluation of PL13-ASO did not show obstruction of SYT2 luminal domain binding in HEK cells with SYT2 stable expression at the cell membrane. (C) PL13 conjugation to *Malat1* ASO payload (approximately 70kDa) showed approximately 80% conjugation efficiency from band intensity measurements with FIJI. (D) Comparison of *Malat1* RNA expression from cells isolated from spinal cord of mice injected with PL13-ASO. PL13 positive cells showed 60.4% decrease in *Malat1* RNA expression compared to PL13 negative cells. Graphical data are expressed in mean±s.e.m., n=3 and was T-test analyzed; ****$p<0.001$, ***$p<0.01$, **$p<0.05$, *$p\leq0.1$
